# An mHealth Intervention Promoting Physical Activity and Healthy Eating in a Family Setting (SMARTFAMILY): Randomized Controlled Trial

**DOI:** 10.2196/51201

**Published:** 2024-04-26

**Authors:** Kathrin Wunsch, Janis Fiedler, Sebastian Hubenschmid, Harald Reiterer, Britta Renner, Alexander Woll

**Affiliations:** 1 Institute of Sports and Sports Science Karlsruhe Institute of Technology Karlsruhe Germany; 2 Department of Computer and Information Science University of Konstanz Konstanz Germany; 3 Department of Psychology University of Konstanz Konstanz Germany

**Keywords:** mobile app, telemedicine, behavior change, health behavior, family, primary prevention, exercise, diet, food and nutrition, randomized controlled trial, accelerometer, wearable electronic devices, social-cognitive determinants, just-in-time adaptive intervention, digital intervention, mobile phone

## Abstract

**Background:**

Numerous smartphone apps are targeting physical activity (PA) and healthy eating (HE), but empirical evidence on their effectiveness for the initialization and maintenance of behavior change, especially in children and adolescents, is still limited. Social settings influence individual behavior; therefore, core settings such as the family need to be considered when designing mobile health (mHealth) apps.

**Objective:**

The purpose of this study was to evaluate the effectiveness of a theory- and evidence-based mHealth intervention (called SMARTFAMILY [SF]) targeting PA and HE in a collective family–based setting.

**Methods:**

A smartphone app based on behavior change theories and techniques was developed, implemented, and evaluated with a cluster randomized controlled trial in a collective family setting. Baseline (*t*_0_) and postintervention (*t*_1_) measurements included PA (self-reported and accelerometry) and HE measurements (self-reported fruit and vegetable intake) as primary outcomes. Secondary outcomes (self-reported) were intrinsic motivation, behavior-specific self-efficacy, and the family health climate. Between *t*_0_ and *t*_1_, families of the intervention group (IG) used the SF app individually and collaboratively for 3 consecutive weeks, whereas families in the control group (CG) received no treatment. Four weeks following *t*_1_, a follow-up assessment (*t*_2_) was completed by participants, consisting of all questionnaire items to assess the stability of the intervention effects. Multilevel analyses were implemented in R (R Foundation for Statistical Computing) to acknowledge the hierarchical structure of persons (level 1) clustered in families (level 2).

**Results:**

Overall, 48 families (CG: n=22, 46%, with 68 participants and IG: n=26, 54%, with 88 participants) were recruited for the study. Two families (CG: n=1, 2%, with 4 participants and IG: n=1, 2%, with 4 participants) chose to drop out of the study owing to personal reasons before *t*_0_. Overall, no evidence for meaningful and statistically significant increases in PA and HE levels of the intervention were observed in our physically active study participants (all *P*>.30).

**Conclusions:**

Despite incorporating behavior change techniques rooted in family life and psychological theories, the SF intervention did not yield significant increases in PA and HE levels among the participants. The results of the study were mainly limited by the physically active participants and the large age range of children and adolescents. Enhancing intervention effectiveness may involve incorporating health literacy, just-in-time adaptive interventions, and more advanced features in future app development. Further research is needed to better understand intervention engagement and tailor mHealth interventions to individuals for enhanced effectiveness in primary prevention efforts.

**Trial Registration:**

German Clinical Trials Register DRKS00010415; https://drks.de/search/en/trial/DRKS00010415

**International Registered Report Identifier (IRRID):**

RR2-10.2196/20534

## Introduction

### Background

Physical activity (PA) and healthy eating (HE) are protective factors for general health and can also enhance health [1]. In contrast, a lack of PA, too much sedentary behavior (eg, excessive screen time and nonactive media use), and an unhealthy diet are serious concerns and increase the risk of health conditions across all ages [2-5]. Although preschool children seem to show high adherence to PA guidelines [6], they have low adherence to screen time guidelines [7]. Research revealed that children, as they become older, and adolescents do not engage sufficiently in PA [8] and frequently make unhealthy food choices [9,10]. Worldwide, 81% of children and adolescents (and 23% of adults) do not meet recommendations on PA levels and HE, that is, fruit and vegetable intake (FVI) [11]. In this regard, a dose-response relationship (ie, the relationship between the dose of PA and the effect observed) was detected, with even slight increases in PA leading to physiological and psychological health benefits in children and adolescents [12,13] and in adults. Hence, health behavior interventions should aim at sustaining PA and HE levels for those adhering to guidelines or at increasing these behaviors in those who do not adhere to guidelines [7,11]. As longitudinal studies showed that behavioral patterns in childhood and adolescence have a low-to-moderate influence on PA patterns in adulthood [14-19], there is a need for interventions already targeting children and adolescents to promote a sustainable and healthier lifestyle.

To achieve this goal, it is important to recognize that health-related behaviors, such as PA and HE, are influenced by social contexts, such as the family environment, and are shaped by social relationships and connections [20,21]. Leisure time PA of children is, for example, directly linked to parental PA levels [22,23], and the eating behavior of children is dependent on their parents’ food choices [24-26]. Therefore, addressing behavioral changes embedded in social contexts might be a promising approach for facilitating an individual’s behavior change. When focusing on children and adolescents, the most imprinting social context is daily family life. Family meals, for example, are often an important part of everyday life in families, and there is accumulating evidence that this collective behavior is associated with a better overall diet quality and BMI [27-29]. In a similar vein, there is some evidence that family-based PA is positively associated with individual PA levels [30]. It has been shown that supportive interactions within a family and shared values about health behavior affect PA engagement [31] and the eating behavior of children [32]. In addition, results of intervention studies indicate that social support is significantly associated with the continuation of exercise programs [33-37] as well as participation in weight loss interventions [38-40].

Families are difficult to reach with typical in-person interventions, as the daily lives of family members are highly different regarding their content and time schedule. Hence, mobile health (mHealth) technologies might be the means of choice, as they are increasingly used as a delivery mode for health behavior change interventions across different age groups. Specifically, smartphone-based apps offer great promise for enhancing PA and HE as well as making health care more accessible and scalable, more cost-effective, and more equitable [41,42]. Reviews and meta-analyses support the view that app‐based mobile interventions are effective and highly promising for changing PA [36,43] and nutrition behaviors [44], especially when implemented in a family setting, including parental involvement [45]. Moreover, a systematic review of economic evaluations of mHealth solutions found a consistent overall reporting of positive economic outcomes (eg, increase in life-years gained, cost savings, and cost-effectiveness) [46].

Regarding functional principles of such mHealth apps, reviews indicate that the strategies or central *building blocks* of app-based interventions mainly encompassed 4 behavior change technique (BCT) clusters [47], including goal setting, feedback and self-monitoring, information, and social support provision, which coincides with successful conventional individual and group-based interventions [44,48,49]. On the basis of causal analyses, an umbrella review found that the effectiveness of interventions was increased by engaging social support, targeting both PA and HE, and using well-defined or established BCTs. However, because mobile interventions distinguish themselves by being interactive, adaptive, time sensitive, and intraindividually dynamic, more dynamic concepts, including the timing of feedback and tailoring tasks and goals to individual progress and capacities as specified in persuasive technology and gamification approaches, might be essential ingredients of effective and focused mobile interventions [44,50,51].

Moreover, mobile interventions are capable of fulfilling the abovementioned demands to be embedded into a social system so that all members of this system, that is, a family, can simultaneously and collectively take part in an intervention and share their goals and progress. However, currently available apps for health promotion are almost exclusively tailored to an individual level [52]. Motivation for behavior performance is higher when the individual is embedded in a social system of mutual appreciation and importance, according to self-determination theory (SDT) [53,54], which was successfully used in PA interventions by enhancing autonomous motivation and fulfilling the 3 basic psychological needs: “autonomy,” “competence,” and “relatedness” [53]. As healthy or unhealthy behavioral patterns are developed and maintained in social contexts, embedding a mHealth intervention in a family setting and targeting all family members might be promising and corresponds to assumptions of family-as-systems approaches [55]. An umbrella review about digital interventions for health behavior change in PA and HE found a lack of studies focusing on the social contexts, while BCTs and a theoretical foundation were associated with higher intervention effectiveness [56]. Another review of the effect of family-based mHealth interventions found heterogeneous results in a limited research body and called for more experimental studies in this area [57]. Overall, there is a lack of randomized controlled mHealth studies that use important key facets of effective interventions such as BCTs and a theoretical foundation while considering the social system of participants.

### Objectives

This study aimed to evaluate the effectiveness of a mobile stand-alone smartphone intervention app that incorporates the family as a social system of high relevance for its single members. We designed and evaluated a multicomponent mHealth intervention that aimed to improve PA and HE in families. The development of the app was based on behavior change theories, including SDT, and the use of BCTs. The behavior of children and parents was targeted to induce family- and individual-based behavior changes. Specifically, family members were using the SMARTFAMILY (SF) app individually and cooperatively. The complete study protocol and more details are available in the study by Wunsch et al [58]. It was hypothesized that the mHealth intervention would positively influence PA variables (steps and moderate to vigorous PA [MVPA]) and the HE variable (FVI) in the whole family.

## Methods

### Study Design

**Figure 1:**
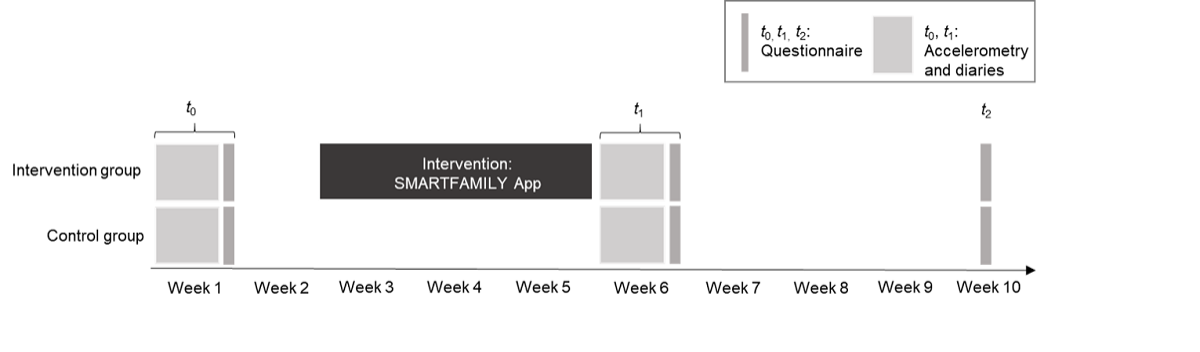
Detailed study design.

The study was conducted and described according to the corresponding study protocol and the CONSORT-EHEALTH (Consolidated Standards of Reporting Trials of Electronic and Mobile Health Applications and Online Telehealth) checklist that provides guidance for standardized reporting of eHealth and mHealth interventions (Multimedia Appendix 1) [36,41-44,46,53,54,59-66]. This is a cluster randomized controlled trial with two groups: (1) an intervention group (IG) receiving the app intervention and (2) a nonintervention control group (CG) that neither received material nor was contacted during the intervention period. The outline of the SF trial is presented in Figure 1. Assessment of outcomes was completed at baseline (*t*_0_), after the 3-week intervention (or no-intervention) period (postintervention; *t*_1_), and 4 weeks after the measurement (follow-up; *t*_2_). As the study protocol is freely accessible [58], the study design and measurements will only be described very briefly. For a complete overview, please refer to the study protocol.

All eligible members of each family participated in an initial visit to the research facility, where they received instructions on how to use various tools that would be used throughout the study. These included wearable devices called accelerometers, which recorded PA levels, as well as paper diaries for tracking their behavior over time. At the end of this initial phase, participants also answered questions related to their habits and behaviors in the past week, which served as a baseline. This information was shared with those participating in the intervention so they could set family goals for themselves based on this starting point.

Participants assigned to the IG were given specially designed smartphones equipped with our SF app. The app was created in an iterative process, incorporating feedback from both the target audience and experts. The development process also drew upon insights from previous research conducted as part of the SMARTACT project, as well as behavioral theories. The programing of the apps was carried out by the Human-Computer Interaction Workgroup at the University of Konstanz, as a component of the SMARTACT project. Study staff ensured that all participants had access to similar technology by providing them with these phones. Providing additional study smartphones was also recommended by the ethics committee for data security reasons. The study staff also carefully explained the app’s functionality to families, handed out a study manual, and were available to assist with any issues that arose. In addition, the accelerometers worn by participants were synced wirelessly with the smartphones through Bluetooth low-energy connections.

When the 3-week intervention began, families in the IG were instructed to establish collective weekly objectives for both PA and HE. The 3-week period was chosen due to practical concerns, as we examined families in their natural setting. In Germany, a continual school period lasts a maximum of 6 to 8 weeks, followed by a vacation period. To conduct the core assessments, including pre- and posttesting accelerometry during 1 continual school period, an intervention period >3 weeks was not feasible for the study design. Longer intervention periods would inevitably mean that there is a cofounding between the assessment period (school time vs vacation). Specifically, they aimed to accumulate a certain number of steps, engage in appropriate amounts of moderate or vigorous PA, plan enjoyable family activities together, and consume adequate portions of fruits and vegetables as well as joint family meals. Families were instructed to formulate the goals together based on their previous performance from the baseline assessments. The goals were set on 1 smartphone for the whole family, and the app notified the family every Sunday to set new goals. Participants were encouraged in the initial explanation phase by the study staff to strive for progressively more ambitious but realistic goals based on their previous behavior. As part of the program, participants revisited and adjusted their goals regularly to keep challenges manageable while promoting continuous improvement. All smartphones were retrieved after the intervention period ended. Therefore, the IG had neither access to the app during *t*_1_ nor afterward.

The app included 10 BCTs [47] for the IG and no BCT for the CG. Intervention BCTs were behavioral goal setting, prompt review of behavioral goals, prompt self-monitoring of behavior, providing feedback on performance, planning social support or social change, prompt identification as a role model or position advocate, setting graded tasks, shaping, prompt rewards contingent on effort or progress toward behavior, and providing rewards contingent on successful behavior.

Once the intervention or control period ended, participants underwent 2 more testing sessions (*t*_1_ and *t*_2_). Our investigation used a single-blind design, and the survey instruments were evaluated for user-friendliness and technical reliability beforehand.

### Eligibility Criteria

Households comprising at least 1 adult caregiver and 1 child aged >10 years residing together could participate in the research. If applicable, other siblings were welcome to join the project as well. Everyone taking part had to possess basic proficiency in handling mobile devices, be physically able to participate in PA independently, and communicate effectively in German. However, exceptions might have been made for younger siblings who met this requirement.

### Ethical Considerations

Before commencement, participants (including minors), parents, or legal guardians signed informed consent forms. Full ethics approval was granted by the University of Konstanz and the Karlsruhe Institute of Technology. This research adhered to the guidelines outlined in the Helsinki Declaration. Furthermore, during the testing sessions, ethical standards were upheld, and personal data were protected.

Families who completed the study were eligible to participate in a prize draw at the end of the study, having the chance to win one of three family-tickets for a big theme park in Germany.

### Randomization and Blinding

This study used a cluster randomized controlled design comparing 2 distinct groups: an IG that received the intervention and a CG that did not receive any treatment. Consenting families were randomly assigned to either arm through a straightforward allocation scheme suitable for cluster trials [60]. While members of the IG were aware of the mHealth aspects of the study, participants in the CG were merely informed about contributing to an epidemiological examination of PA and overall health. Accordingly, all participants wore accelerometers for 1 week twice across a 10-week duration to ensure accurate and dependable measurements. In addition, participants completed various questionnaires during this period.

### Participants

Participants were recruited in schools, school holiday programs, music schools, and sports clubs via personal communication, newspapers, social media, and email distribution lists of the Karlsruhe Institute of Technology.

Overall, 48 families (CG: n=22, 46%, with 68 participants and IG: n=26, 54%, with 88 participants) were recruited for the study. Two families (CG: n=1, 2%, with 4 participants and IG: n=1, 2%, with 4 participants) chose to drop out of the study owing to personal reasons before *t*_0_.

### Measurements

#### PA Measures

##### Device-Measured PA (Accelerometry)

Hip-worn (right side) 3-axial accelerometers (Move 3 or Move 4, Movisens GmbH) were used to continuously record PA levels. These accelerometers are scientific research instruments with a measurement range of +16 g to −16 g, an output rate of 64 Hz, physical dimensions of 62.3×38.6×11.5 mm, a weight of 25 g, and custom epoch lengths (ie, 10 s). Data were recorded in a raw format (64 Hz) and were summarized afterward in the epoch lengths of choice. Epoch length was chosen as 10 seconds as shorter epoch lengths are believed to be more appropriate to estimate vigorous PA and assess PA in children owing to intermittent movement behavior [67,68]. Validity has been evaluated for a previous version of the accelerometer (Move 2), which uses comparable digital signal processing as the Move 3 or Move 4 has been considered accurate for assessing steps [69] and energy estimation [70,71] in adults. Handling the accelerometer was explained and demonstrated by a study instructor. Participants were instructed to wear the accelerometer during wake time and remove it only during a shower, swimming, or certain sports involving bodily contact to minimize the probability of injuries. The outcomes for the accelerometer that were used for this study were MVPA (>3.0 metabolic equivalents [MET]) and steps for all participants. MET values were calculated based on activity class (based on acceleration and barometric signals), which determines the estimation model. Afterward, movement acceleration, altitude change, and demographics were combined in the model for the MET estimation [71]. Accelerometer data were included if a minimum wear time of at least 8 hours a day for at least 4 of the 7 days during the measured week was obtained. Nonwear time was calculated in 30-second intervals. The nonwear time detection was based on an algorithm that used accelerometry and temperature signals over a 10-minute window to distinguish between wear time, nonwear time, and sleep, as described elsewhere [72]. For valid measurements, the average of MVPA and steps per valid day was multiplied by 7 to estimate the total minutes per week.

##### Self-Reported PA

At the end of each measurement week, adults were asked to fill short version of the International Physical Activity Questionnaire in German [73], asking retrospectively for activities during the previous week. The results of the question relating to minutes spent in moderate (comprising moderate activity and walking) and vigorous PA were calculated for this study by multiplying the reported number of days with the reported duration of the indicated activity per day and then adding the values of moderate and vigorous PA to MVPA per week. Children completed the 60-Minute Screening Measure [74] for MVPA, which yields the number of days with at least 60 minutes of MVPA according to the (now outdated) World Health Organization guidelines [75]. In addition, a PA diary was used for all participants at *t*_0_ and *t*_1_, which is not included in the current examination owing to noncomparable results [68]. Parents and children were instructed to fill out their self-reported questionnaires and diaries independently.

##### FVI Measures

FVI was assessed using a single item asking for the total amount of fruits and vegetables consumed within a typical week [76] in the questionnaire as well as using a description of detailed food consumption during *t*_0_ and *t*_1_ by indicating the time of the meal, ingredients, portions of FVI, and whether the meal was consumed within the family or alone in a diary.

### Secondary Outcomes

#### Demographics

In the *t*_0_ questionnaire, demographic information of the participants was collected, including sex, age, height, and weight.

#### Health Status

Perceived general health was assessed using a single item [76].

#### Intrinsic Motivation Toward PA

According to the SDT, activity-related self-determination was assessed using the Behavioral Regulation in Exercise Questionnaire (BREQ-2) [77]. BREQ-2 assesses the manifestation of the 5 different regulation modes by the SDT, reflected by the subscales of amotivation (4 items), external (4 items), introjected (3 items), identified (3 items), and intrinsic (4 items) regulations. Responses were made on a 4-point Likert scale, ranging from 0=*not true*, 1=*rather not true*, 2=*rather true* to 3=*true*. For the analysis, a relative autonomy index was formed. It is derived from the subscales and gives an index of the degree to which respondents feel self-determined. The index is obtained by applying a weighting to each subscale and then summing these weighted scores. In other words, each subscale score is multiplied by its weighting, and then these weighted scores are summed. Higher, positive scores indicate greater relative autonomy; lower, negative scores indicate more controlled regulation.

#### Intrinsic Motivation Toward HE

For assessing dietary-related intrinsic motivation, the Regulation of Eating Behavior Scale [78] was used. The dimension “integrated regulation” was omitted, resulting in a total of 5 subscales, coded from 0 to 3. A sum score was built analogous to the BREQ-2.

#### Self-Efficacy for PA and HE

Activity-related self-efficacy and dietary-related self-efficacy were assessed using the health-specific self-efficacy scales, comprising 5 items for each behavior-related dimension [79]. Participants were asked how certain they were to handle different health-specific barriers. Responses were given on a 4-point Likert scale, ranging from 1=very uncertain, 2=uncertain, 3=certain to 4=very certain. A sum score was built for both scales.

#### Family Health Climate

Shared perceptions and cognitions regarding health behaviors were assessed by use of the Family Health Climate (FHC) Scale [80], comprising the 2 separate scales for PA (FHC-PA) and nutrition (FHC Nutrition [FHC-NU]). FHC-PA contains 14 items, which are assigned to the 3 subscales of value (5 items), cohesion (5 items), and information (4 items). FHC-NU includes 17 items, comprising the 4 subscales of value (4 items), cohesion (5 items), consensus (3 items), and communication (5 items). Responses for each dimension were scored on a 4-point Likert scale ranging from 0=*not true*, 1=*rather not true*, 2=*rather true* to 3=*true*. Sum scores were built for both scales.

#### Joint PA and Meals Within the Family

Joint PA and nutrition were assessed using a single item that referred to the number of activities and meals in which the whole family was involved during the past week. The mean value per family was used for the analysis.

### Statistical Analysis

The analyses were run with different packages of R (R Foundation for Statistical Computing) [81] and RStudio (Posit) [82]. The package *ggplot2* was used for visualizations [83] following the instructions by Allan et al [84]. Mixed models were calculated using the package *lmerTest* [85] with participants (level 1) nested in families (level 2) to acknowledge the hierarchical structure of the data. The result tables of the regression analyses were generated using the package *sjPlot* [86]. In total, 6 final models were calculated, 1 with each measurement method and outcome parameter (1 steps, 3 MVPA, and 2 FVI intake per wk) as dependent variables. Assumptions were checked using the visualization of the *performance* package [87]. A hierarchical approach was used for the inclusion of the control variables, and the model fit was assessed with the Akaike information criterion for sensitivity analysis. In addition, 2 models were calculated for joint PA and nutrition based on the family mean values without the random factor family because it was defined as “all family members were present.”

The predictor group (ie, control=0 and intervention=1)×time (dummy coded with *t*_0_ as reference for *t*_1_ and *t*_2_) was included in the models to evaluate the interaction effect (main effect) of the intervention on the 8 outcome parameters. To assess sensitivity regarding the additional variables, the secondary outcome parameters self-efficacy, intrinsic motivation, and the FHC were added either referring to PA or FVI depending on the outcome, and the control variables health status, population (adult=0 and children=1), sex (0=male and 1=female), and nonwear time per week—only for the device-measured PA models—were tested for the inclusion in the random effect models. In addition, the inclusion of random slopes and random intercepts were evaluated based on the model fit. The level of statistical significance was set a priori to α<.05 with no correction for multiple comparisons.

## Results

### Data Availability and Participant Characteristics

In total, 46 families with 148 participants (74/148, 50% adults: 45/74, 61% female and 29/74, 59% male; and 74/148, 50% children: 38/74, 51% female and 36/74, 49% male) participated in the study. The mean ages of adults and children were 47.8 (SD 5.0) and 13.3 (SD 2.7) years, while the average BMI values were 24.8 (SD 4.1) and 19.0 (SD 3.3), respectively. Testing took place between December 1, 2017, and January 31, 2020. Technical issues with the app during the intervention, insufficient wear time of the accelerometer (ie, <4 days with >8 hours wear time), and missing data for the self-reported items led to the inclusion of a differing number of participants for each calculated model (depending on the outcome variables; Multimedia Appendices 2 and 3). Participant characteristics of the 46 families separated by group (CG vs IG), population (children vs adults), and sex (male vs female) are displayed in Table 1. Descriptive results for all included outcomes and predictors can be found in Table 2.

**Table 1:** Participant characteristics of the 46 families included in the SMARTFAMILY trial.

Characteristics		Control group			Intervention group	
		Children (n=32)	Adults (n=32)	Children (n=42)		Adults (n=42)
**Sex, n (%)**						
	Male	15 (47)	11 (34)	21 (50)		18 (43)
	Female	17 (53)	21 (66)	21 (50)		24 (57)
**Age (y), mean (SD)**						
	Male	13.1 (3.23)	49.1 (5.02)	13.4 (2.48)		50.2 (5.58)
	Female	13.4 (2.61)	47.0 (3.90)	13.5 (2.58)		46.1 (4.70)
**BMI (kg/m^2^), mean (SD)**						
	Male	18.7 (2.59)	25.7 (3.30)	19.0 (4.06)		26.6 (5.35)
	Female	19.2 (3.14)	24.7 (3.63)	18.9 (3.00)		23.3 (3.01)

**Table 2:** Descriptive results of the 46 families included in the SMARTFAMILY trial. Data were assessed for the three measurement weeks: baseline (*t*_0_), after intervention (*t*_1_), and 4 weeks after intervention at follow-up (*t*_2_).

Measurements	Control group, mean (SD)								Intervention group, mean (SD)							
	*t*_0_		*t*_1_			*t*_2_			*t*_0_			*t*_1_			*t*_2_	
	Child	Adult	Child	Adult	Child		Adult	Child		Adult	Child		Adult	Child		Adult
Steps (counts/wk)	62,800 (21,800)	60,000 (24,000)	58,900 (23,600)	59,500 (27,700)	—^a^		—	66,200 (22,400)		66,700 (24,100)	64,000 (24,600)		66,500 (32,800)	—		—
MVPA^b^ (min/wk)	721 (303)	674 (282)	652 (303)	675 (338)	—		—	711 (307)		745 (237)	609 (294)		716 (316)	—		—
Nonwear time (min/wk)	4660 (789)	4010 (389)	4910 (677)	4140 (537)	—		—	4720 (538)		4120 (618)	4540 (596)		4190 (731)	—		—
IPAQ^c^ MVPA (min/wk)	X^d^	975 (916)	X	826 (1010)	X		772 (737)	X		1060 (1030)	X		1080 (839)	X		999 (678)
KIKA^e^ MVPA (active d/wk)	4.46 (1.67)	X	3.92 (1.52)	X	4.25 (1.68)		X	4.19 (1.86)		X	4.00 (1.76)		X	3.57 (1.87)		X
FVI^f^ quest (portions/wk)	10.2 (8.94)	12.3 (6.54)	9.77 (8.01)	12.5 (8.13)	8.84 (6.82)		13.5 (7.88)	14.8 (11.0)		16.4 (9.77)	14.4 (9.29)		18.2 (11.3)	23.7 (55.9)		17.3 (10.9)
FVI diary (portions/wk)	12.2 (5.73)	14.3 (8.14)	11.3 (8.00)	15.0 (9.77)	—		—	17.1 (12.0)		16.2 (8.80)	17.3 (11.2)		20.3 (11.6)	—		—
Health	4.09 (0.86)	3.74 (0.73)	4.07 (0.77)	3.61 (0.74)	3.96 (0.82)		3.74 (0.76)	4.21 (0.58)		3.95 (0.70)	4.14 (0.65)		3.94 (0.64)	4.25 (0.57)		3.85 (0.62)
M intrinsic NU^g^ (RAI)^h^	24.9 (16.1)	23.6 (17.7)	21.5 (14.3)	25.9 (14.9)	21.1 (14.9)		24.0 (18.2)	22.8 (16.4)		29.8 (12.6)	27.0 (17.6)		32.2 (14.3)	25.9 (16.3)		31.7 (13.8)
M intrinsic PA^i^ (RAI)	38.1 (17.7)	36.4 (16.9)	35.8 (17.2)	33.6 (15.6)	36.1 (14.4)		32.5 (16.7)	39.2 (13.7)		39.0 (15.5)	40.0 (14.5)		39.1 (12.4)	38.8 (15.2)		39.3 (13.7)
FHC^j^ NU^k^	30.3 (6.85)	32.1 (8.83)	27.9 (7.82)	29.1 (7.48)	28.4 (7.77)		30.0 (8.82)	30.9 (8.64)		34.9 (6.81)	31.6 (8.38)		35.1 (7.72)	31.7 (8.52)		36.0 (6.87)
FHC PA^l^	20.9 (7.15)	22.2 (7.18)	20.2 (5.83)	19.5 (5.77)	21.6 (6.88)		19.5 (7.32)	22.8 (7.63)		25.5 (7.84)	22.3 (7.82)		23.4 (8.31)	23.2 (6.55)		24.2 (7.85)
Self-efficacy NU (RAI)	49.0 (10.1)	42.9 (11.7)	48.1 (7.86)	49.1 (9.60)	49.2 (10.3)		46.2 (10.8)	47.7 (12.5)		49.8 (11.4)	47.2 (14.8)		49.9 (10.2)	50.2 (11.5)		50.1 (10.3)
Self-efficacy PA (RAI)	50.9 (9.32)	44.8 (8.39)	50.6 (9.03)	43.7 (9.51)	51.5 (10.6)		45.4 (9.15)	49.4 (9.38)		47.8 (11.9)	49.7 (9.08)		49.6 (12.1)	49.9 (10.7)		51.3 (10.9)
Joint PA	0.586 (0.907)	0.429 (0.634)	0.556 (0.641)	0.815 (1.14)	0.690 (0.930)		0.520 (0.586)	0.946 (1.31)		0.917 (1.42)	0.972 (0.910)		0.719 (0.772)	1.00 (1.00)		0.935 (0.929)
Joint NU	8.97 (5.28)	7.55 (4.20)	7.67 (4.10)	7.39 (3.57)	7.14 (4.03)		8.39 (4.46)	7.97 (4.30)		8.45 (4.08)	6.95 (3.69)		7.21 (3.86)	7.94 (4.56)		8.03 (4.76)

^a^Not measured at *t*_3_.

^b^MVPA: moderate to vigorous physical activity.

^c^IPAQ: International Physical Activity questionnaire.

^d^The International Physical Activity questionnaire was used for adults, and the 60-Minute Screening Measure was used for children.

^e^KIKA: 60-Minute Screening Measure.

^f^FVI: fruit and vegetable intake.

^g^M intrinsic NU: intrinsic motivation toward nutrition.

^h^RAI: relative autonomy index.

^i^M intrinsic PA: intrinsic motivation toward physical activity.

^j^FHC: Family Health Climate.

^k^NU: nutrition.

^l^PA: physical activity.

All control variables (except sex for the PA questionnaire of children) improved the model fit based on the Akaike information criterion and were therefore included in the final sensitivity models. Random slopes were not supported by the data, but random intercepts were used for all models. Sensitivity analysis showed no difference in patterns for the effectiveness of the intervention (Tables S1-S6 in Multimedia Appendix 3). Therefore, only the main models are reported. Data and code are available at the Open Science Framework [88].

### Effect of the Intervention on PA

**Figure 2:**
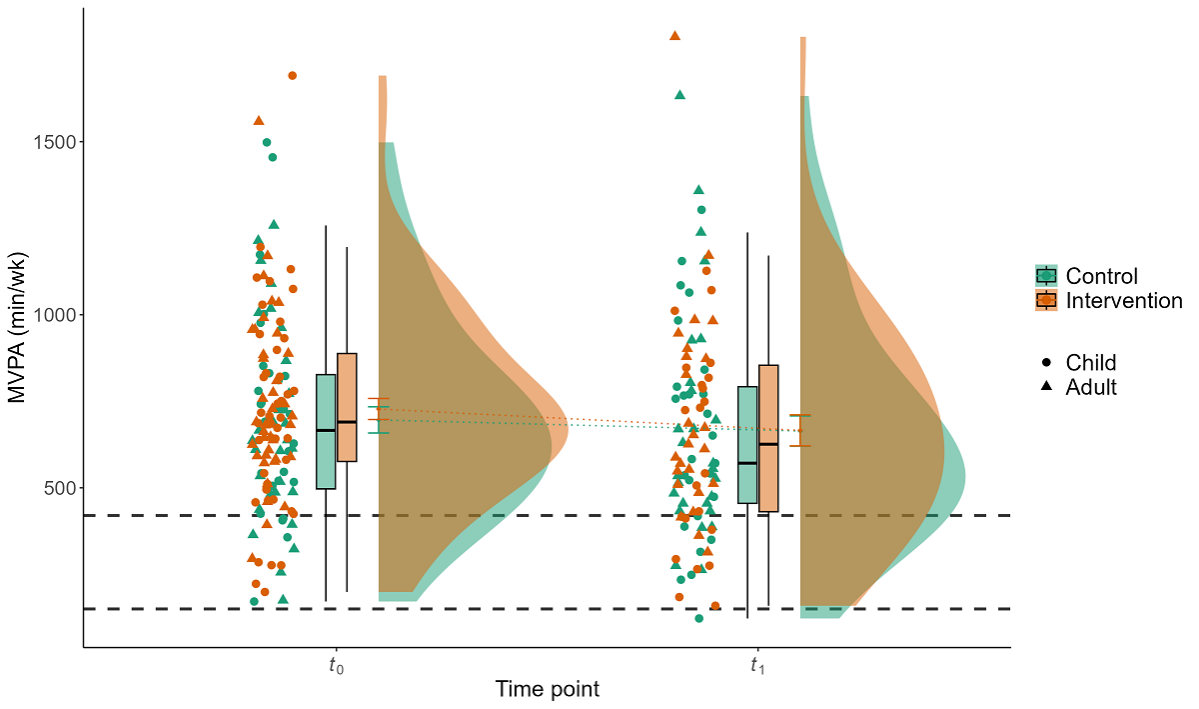
Interaction effect of group×time for device-measured physical activity (PA) for the parameter minutes of moderate to vigorous PA (MVPA) per week. Displayed are the mean MVPA (y-axis) of 109 participants during 1 week of baseline measurement (*t*_0_) and 1 week of postintervention measurement after a 3-week intervention and waiting period (*t*_1_) for the control group (green) and the intervention group (red), stratified by children and adults. The gray dashed lines represent the PA recommendations for children (420 min/wk) and adults (150 min/wk).

**Figure 3:**
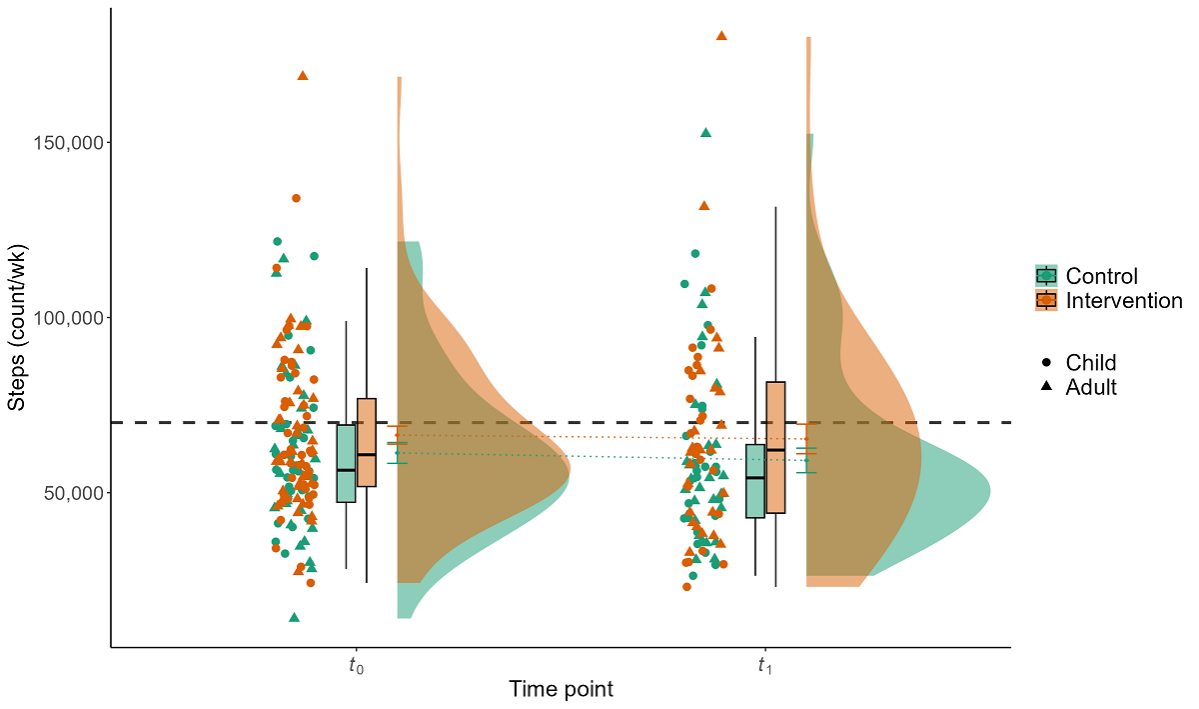
Interaction effect of group×time for device-measured physical activity for the parameter steps per week (steps). Displayed are the mean step count (y-axis) of 109 participants during 1 week of baseline measurement (*t*_0_) and 1 week of postintervention measurement after a 3-week intervention and waiting period (*t*_1_) for the control group (green) and the intervention group (red), stratified by children and adults. The gray dashed line represents the commonly used step recommendation of 10,000 steps per day (70,000 steps/wk).

Results of the multilevel models indicate no significant main effect for the interaction of the group with time in the PA outcomes (Tables S1-S4 in Multimedia Appendix 2). The only significant main effect was found between *t*_0_ and *t*_1_ for the self-reported 60-Minute Screening Measure in children (*P*=.03; β=−.35). Figures 2 and 3 display the descriptive results of the main effects for device-measured PA outcomes, MVPA, and step count. As displayed by the gray dotted lines in Figure 2, both mean and median values are clearly above the recommendation for MVPA [89] for both children (ie, 60 min/d on average, 420 min/wk) and adults (ie, 150 min/wk) in both groups and at both measurement periods. For steps, mean and medium values are shortly below the commonly used 10,000 steps per day goal [90,91] for all participants (Figure 3).

### Effect of the Intervention on FVI

**Figure 4:**
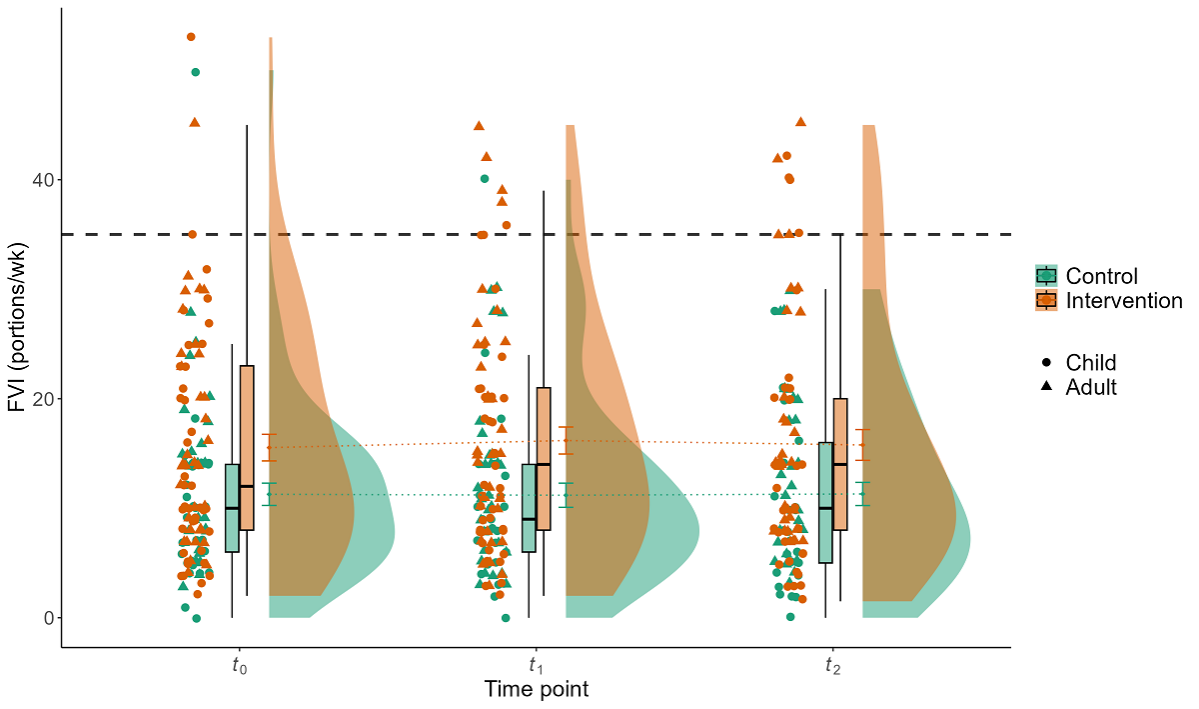
Interaction effect of group×time for the parameter fruit and vegetable intake (FVI) per week assessed by questionnaire. Displayed are the mean FVI (y-axis) of 118 participants related to the week of baseline measurement (*t*_0_), the week of postintervention measurement after a 3-week intervention and waiting period (*t*_1_), and the week of follow-up measurement (*t*_2_) for the control group (green) and the intervention group (red), stratified by children and adults. The gray dashed line represents the recommendation for daily FVI of 5 portions (35 portions/wk).

Results of the multilevel models indicate no significant interaction of group×time concerning FVI (Tables S5 and S6 in Multimedia Appendix 2). The only observed significant main effect was a group effect for FVI from the questionnaire (*P*=.049; β=.23). Figure 4 displays the descriptive results for self-reported FVI per week assessed by the questionnaire. Here, both mean and median values are clearly below the recommended FVI of 35 portions per week [92].

### Effect of the Intervention on Joint PA and Meals

Results of the linear model indicate no significant effect for group×time but a significant main effect for group (*P*<.001; β=.25) in joint PA. For joint meals, a significant effect for group× time (*t*_2_) was observed (*P*=.04; β=.16). In addition, a significant reduction of joint meals between *t*_0_ and *t*_1_ was observed (*P*=.001; β=−.31). All results are displayed in Tables S7 and S8 in Multimedia Appendix 2.

## Discussion

### Principal Findings

The SF trial evaluated the effectiveness of a mHealth intervention to increase PA and HE in families. Extending previous research, the behavior of children and parents was targeted to induce individual behavior changes that are anchored in daily family life. Moreover, besides a theoretical foundation, several BCTs were included, which contribute to the fulfillment of basic psychological needs according to the SDT [53,54]. Overall, no evidence for meaningful and statistically significant increases in PA and HE levels of the intervention was observed in our physically active sample. Guidelines for weekly MVPA were highly over exceeded in this study sample. However, PA levels were sustained and did not decrease, independent of group affiliation. Intervention effectiveness will be re-evaluated in the SMART*FAMILY2.0* trial, which includes gamification features, health literacy, a just-in-time adaptive intervention, and more sophisticated app features.

### PA and HE

Neither hypotheses, that PA or HE would increase as a result of the intervention, were supported by any self-reported or device-based measure. This is in line with previous digital health studies that also found heterogeneous results, with a majority of studies, however, revealing at least some significant benefit of interventions [44,56]. However, it needs to be noted that the current sample was exceedingly high in their amount of PA, which might have impacted the current null findings. Regarding PA, participants exceeded the recommended amount of 420 minutes of MVPA per week for children and adolescents by 230 minutes on average but fell short of the recommended amount of 35 portions of fruits and vegetables by 21 portions. Although the current sample can be classified as being highly active in a national and international comparison [93], they cannot be classified as equally healthy regarding their eating behavior. In a German-wide representative sample, 17% of 6- to 11-year-olds and 20% of 12- to 17-year-olds reached the recommendation on fruit consumption, 7% and 23%, respectively, for vegetable consumption [94]. Comparable studies are rare. A systematic review and meta-analysis of the efficacy of mHealth interventions to improve PA only focused on inactive participants, as the largest effects are to be expected in this group [95]. In the review [95], only 2 studies were found that implemented a stand-alone mHealth intervention. However, the results were in favor of the interventions for inactive participants. Another review found only 50% of mHealth interventions to be effective [96].

Another impacting factor in our investigation might have been that the intervention duration was rather short; however, it was determined by the short intervals between school holidays, which were omitted within the measurement periods. Hence, the intervention was only carried out within school periods to depict “normal” days. The structured-day hypothesis postulates that health behaviors differ between structured days and school vacation times or weekends. A meta-analysis has shown that the largest effects can be expected for interventions that last over several weeks [97], which might have had a positive influence on intervention effectiveness. However, mHealth intervention studies even revealed significant behavior change effects with intervention durations of only 1 [98], 2 [99], and 3 weeks [100]. These results collectively suggest that both health behaviors should be considered separately but can be addressed together in interventions to harness their synergistic impact on health, a notion supported by other studies as well [101,102]. Although not everyone might need an intervention in both factors, few fulfill both PA and FVI guidelines.

### Family-Based Interventions

Studies focusing on mHealth family-based interventions are rare, especially those including randomized controlled trials. Results for parent- and child-based digital interventions are heterogeneous regarding their reported effectivity, often combined with nondigital interventions, and focused on the behavior of the children instead of the behavior of the whole family [103,104]. A recent review pointed to the effectiveness of digital interventions for obesity prevention, including the enhancement of HE and PA in children, but only 2 of the included studies focused on mHealth interventions [105]. The first study that focused on children with obesity used an app for self-monitoring of weight and goal setting, which led to a greater reduction of weight than the standard care group after 6 months [106]. The other study aimed to improve fundamental movement skills in 3- to 6-year-old children and found an improvement in these skills after a 2-month intervention period compared to a CG [107]. Of the 7 studies included in another review [57], 2 pilot studies reported significant improvements in PA; 3 studies found evidence for some improvements in PA measures, for example, collaborative PA of children and parents; and the remaining 2 studies found no evidence for an effect. Interestingly, 1 study pointed out that the adolescent dropout rate was 12.2 times higher if their parents stopped using the app.

In this regard, analyzing family behavior and dyadic relationships will be a promising approach for future investigations. Studies suggest that family meal practices and values can support HE [108] and that the frequency of shared family meals is significantly related to nutritional health in children and adolescents [27-29]. The results of this study do not support the assumption that joint PA or joint meals (ie, some type of “quality time” within the family) impacts PA or HE behavior. However, it needs to be acknowledged that joint PA was rather low in our sample (only about 1 joint activity per wk).

In summary, the results of this study point to an urgent need to better understand intervention engagement, both quantitatively and qualitatively, to design effective interventions [109,110]. If researchers have a better idea about what works when and for whom, then mHealth interventions can be tailored more specifically and facilitate the benefit of individualization, which was found to be related to enhanced effectiveness [51]. This knowledge can be used for just-in-time adaptive interventions, which aim to support in the exact moment when support is needed and behavior change is realistic [50,111]. Overall, research on mHealth interventions for families remains limited, particularly in the realm of primary prevention. However, theoretical frameworks emphasize the significance and potential of such interventions for promoting PA and HE [56,105]. An important consideration for designing future evaluations of interventions is to additionally account for methods that go beyond pre-post follow-up designs to account for the timelines and complexity of mHealth interventions on a minute-to-minute basis. This would allow important insights for the analysis of continuous longitudinal data in ambulatory assessment studies [112]. In addition, future studies would benefit from recruiting participants with more diversity in their activity and BMI levels and from either restricting the age of children and parents more clearly or tailoring the mHealth intervention specifically to the age of participants [51] to factor the different needs, for example, while growing up [113]. It needs to be noted that already active participants are still a valid target for interventions to maintain a sufficient PA level throughout life. Future primary prevention–focused interventions should, however, clearly define a PA level–specific aim. An example would be to aim for PA maintenance in sufficiently active participants and PA increase in insufficiently active ones and to frequently evaluate PA levels to allow for early interventions. However, both strategies need different accompanying BCTs (adjusted for maintenance purposes), which should also be accounted for.

### Strengths and Limitations

The main strengths of the SF intervention are as follows: it collaboratively targets the family, it is designed as a cluster randomized controlled trial, it is theory based, and it incorporates 10 different BCTs. Furthermore, the goal-setting feature in the app is ad libitum selected by the family to fit their schedule and preferences with guidance from the results of the first measurement week. This enables the families to set self-selected goals, which have been found to increase motivation and adherence [114,115]. Another strength regarding the evaluation is that multiple outcome measures of self-reported and device-measured PA were considered. This is important as these measures are known to yield different results, and including multiple measurement tools improves the plausibility of the results [68]. Finally, using advanced statistical methods to consider the nested structure of the data by applying multilevel analyses enhanced the accuracy of the results, as it considered the variance based on the clustering of participants in families.

However, some limitations must be acknowledged. The actual sample size differed from the planned size by 6 families and 8 participants, falling short of the intended 52 families and 156 participants as determined by the power analysis in the study protocol [58]. This varied from analysis to analysis owing to missing values in distinct variables. In addition, the total population of the IG and CG were not perfectly balanced, and the level of significance was not adjusted for multiple primary outcomes. However, the observed *P* values for the interactions in this paper are not close to .05, except for the group×time point interaction for joint meals per week and joint physical activities. Therefore, the abovementioned issues have likely no large impact on the study results. Regarding our sample, family sizes and ages within families have been very diverse. However, there is a lack of knowledge about how these composite family structures influence results regarding behavior change or the accomplishment of healthy lifestyles. For example, it might be assumed that older parents might be more aware of healthy food choices, as they consider healthy nutrition as being more important for themselves than their younger counterparts [116]. Advanced paternal age is associated with an increased risk of eating disorders in children, whereas younger paternal age is associated with a decreased risk of eating disorders in children [117]. A further restriction might be the age range, especially for children and adolescents. As this study includes the whole family, children of different ages and with different needs and perceptions were addressed similarly by the app, which might have affected intervention effects. Future interventions should aim to address the individual needs of the family members and tailor the intervention more specifically to the participants. Regarding the assessment of theoretical assumptions, it would have been interesting if the included influenced the effectiveness of our intervention. This cannot be answered by this study, as we only assessed the influence of covariables on PA and not on the effectiveness. Hence, the key constructs of the SDT [53,54] might separately be assessed in future studies. Another important factor concerning the sample of this study, which could explain the lack of significant effects for PA, was probably the highly active sample with approximately 650 minutes of MVPA per week per person, which already fulfilled the guidelines of the World Health Organization for PA [118]. This is in contrast to recent research about PA guideline fulfillment, which reliably reveals only small proportions of participants fulfilling the PA recommendations, with lower amounts corresponding to increasing ages [8,119]. Hence, future interventions should either aim at recruiting inactive participants to be able to detect changes in PA or should adjust statistical analyses and intervention design for monitoring PA maintenance over time. Our sample also deviated from the general German population regarding body composition and HE behavior [94]. With BMIs between 18 and 19 in children, and between 23 and 26 in adults, our study participants were of normal weight. Studies have shown that intervention effects can be expected to be higher on participants with overweight or obesity, both for HE and PA interventions [120]. This might be a probable explanation for the absence of intervention effects (ie, there was no or only little room for improvement in our sample). An additional aspect was that the participants had to use the provided smartphones instead of their own for equality and data security reasons, which can be burdensome. However, previous research showed no differences in engagement between participants with their own smartphones versus additional smartphones [121]. If a program aims for long-term sustainability beyond a scientific scope, the use of an additional phone must be considered very carefully. Another potentially limiting factor is the comparably short duration of the intervention. On the basis of literature regarding behavior change theories (ie, the transtheoretical model) [122,123], an intervention duration of 3 weeks might not have been sufficient [44], as the largest effects are expected for interventions that last over several weeks [97] in behavioral studies. However, mHealth intervention studies even revealed significant behavior change effects with intervention durations of only 1 [98], 2 [99], and 3 weeks [100]. In a similar vein, a recent meta-analysis of mobile apps for diet showed that interventions with longer duration were not generally more effective [44].

### Conclusions

Taken together, the evaluation of the SF trial expands the existing body of evidence as it investigated the influence of a theory-based mHealth intervention targeting PA and HE in a collective family–based setting. However, no significant evidence for the effectiveness of the trial has been found. This finding, however, is not unique to mHealth interventions [56] and might be attributable to an initially active sample with a normal BMI. Therefore, future evaluations of interventions should also (1) consider methods that go beyond pre-post follow-up designs to account for the timeliness and complexity of mHealth interventions, (2) consider recruiting participants of all activity and weight levels, and (3) control for or restrict ages of children and parents.

## Appendix

Multimedia Appendix 1 CONSORT-EHEALTH (Consolidated Standards of Reporting Trials of Electronic and Mobile Health Applications and Online Telehealth) checklist.

Multimedia Appendix 2 Detailed results of the main analyses.

Multimedia Appendix 3 Detailed results of the sensitivity analyses.

## Abbreviations

BCT: behavior change technique
BREQ-2: Behavioral Regulation in Exercise Questionnaire
CG: control group
CONSORT-EHEALTH: Consolidated Standards of Reporting Trials of Electronic and Mobile Health Applications and Online Telehealth
FHC: Family Health Climate
FHC-NU: Family Health Climate–Nutrition
FHC-PA: Family Health Climate–Physical Activity
FVI: fruit and vegetable intake
HE: healthy eating
IG: intervention group
MET: metabolic equivalents
mHealth: mobile Health
MVPA: moderate to vigorous physical activity
PA: physical activity
SDT: self-determination theory
SF: SMARTFAMILY
