# Supplementary material for: An mHealth Intervention Promoting Physical Activity and Healthy Eating in a Family Setting (SMARTFAMILY): Randomized Controlled Trial
Source: JMIR Mhealth Uhealth. 2024 Apr 26;12:e51201. doi: 10.2196/51201 (PMC11087865; doi:10.2196/51201)
Supplement: Multimedia Appendix 1 [file mhealth_v12i1e51201_app1.docx]

# CONSORT-EHEALTH (V 1.6.1) - Submission/Publication Form

**Your name ***

First Last

Kathrin Wunsch

**Primary Affiliation (short), City, Country ***

University of Toronto, Toronto, Canada

Karlsruhe Institute of Technology (KIT), Karlsruhe, Germany

**Your e-mail address ***

abc@gmail.com

Kathrin.Wunsch@kit.edu

**Title of your manuscript ***

Provide the (draft) title of your manuscript.

# SMART*FAMILY*: A randomized-controlled trial as a first approach to designing mobile health interventions to promote physical activity and healthy eating in a family setting

**Name of your App/Software/Intervention ***

If there is a short and a long/alternate name, write the short name first and add the long name in brackets.

SMARTFAMILY

**Evaluated Version (if any)**

e.g. "V1", "Release 2017-03-01", "Version 2.0.27913"

SF (version 1)

**Language(s) ***

What language is the intervention/app in? If multiple languages are available, separate by comma (e.g. "English, French")

German

**URL of your Intervention Website or App**

e.g. a direct link to the mobile app on app in appstore (itunes, Google Play), or URL of the website. If the intervention is a DVD or hardware, you can also link to an Amazon page.

<https://www.uni-konstanz.de/smartact/>

**URL of an image/screenshot (optional)**

http://www.sport.kit.edu/smartfamily/

**Accessibility ***

Can an enduser access the intervention presently?

access is free and open

access only for special usergroups, not open

access is open to everyone, but requires payment/subscription/in-app purchases

app/intervention no longer accessible

Others:

**Primary Medical Indication/Disease/Condition ***

e.g. "Stress", "Diabetes", or define the target group in brackets after the condition, e.g. "Autism (Parents of children with)", "Alzheimers (Informal Caregivers of)"

Target group are healthy families.

**Primary Outcomes measured in trial ***

comma-separated list of primary outcomes reported in the trial

Device-based physical activity via accelerometers, self-reported physical activity levels via questionnaire and diary, fruit and vegetable intake via questionnaire and diary.

**Secondary/other outcomes**

Are there any other outcomes the intervention is expected to affect?

In this first trial, there are no secondary outcomes of interest.

**Recommended "Dose" ***

What do the instructions for users say on how often the app should be used?

Approximately Daily

Approximately Weekly

Approximately Monthly

Approximately Yearly

"as needed"

Others: several times daily

**Approx. Percentage of Users (starters) still using the app as recommended after 3 months ***

unknown / not evaluated

0-10%

11-20%

21-30%

31-40%

41-50%

51-60%

61-70%

71%-80%

81-90%

91-100%

Others: app is only available for three weeks during intervention period.

**Overall, was the app/intervention effective? ***

yes: all primary outcomes were significantly better in intervention group vs control

partly: SOME primary outcomes were significantly better in intervention group vs control

no statistically significant difference between control and intervention

potentially harmful: control was significantly better than intervention in one or more outcomes

inconclusive: more research is needed

Others: Data was not analyzed yet.

**Article Preparation Status/Stage ***

At which stage in your article preparation are you currently (at the time you fill in this form)

not submitted yet - in early draft status

not submitted yet - in late draft status, just before submission

submitted to a journal but not reviewed yet

submitted to a journal and after receiving initial reviewer comments

submitted to a journal and accepted, but not published yet

published

Others:

**Journal ***

If you already know where you will submit this paper (or if it is already submitted), please provide the journal name (if it is not JMIR, provide the journal name under "other")

not submitted yet / unclear where I will submit this

Journal of Medical Internet Research (JMIR)

JMIR mHealth and UHealth

JMIR Serious Games

JMIR Mental Health

JMIR Public Health

JMIR Formative Research

Other JMIR sister journal

Others:

**Is this a full powered effectiveness trial or a pilot/feasibility trial? ***

Pilot/feasibility

Fully powered

**Manuscript tracking number ***

If this is a JMIR submission, please provide the manuscript tracking number under "other" (The ms tracking number can be found in the submission acknowledgement email, or when you login as author in JMIR. If the paper is already published in JMIR, then the ms tracking number is the four-digit number at the end of the DOI, to be found at the bottom of each published article in JMIR)

no ms number (yet) / not (yet) submitted to / published in JMIR

Others:

## TITLE AND ABSTRACT

**1a) TITLE: Identification as a randomized trial in the title**

**1a) Does your paper address CONSORT item 1a? ***

I.e does the title contain the phrase "Randomized Controlled Trial"? (if not, explain the reason under "other")

yes

Others:

**1a-i) Identify the mode of delivery in the title**

Identify the mode of delivery. Preferably use “web-based” and/or “mobile” and/or “electronic game” in the title. Avoid ambiguous terms like “online”, “virtual”, “interactive”. Use “Internet-based” only if Intervention includes non-web-based Internet components (e.g. email), use “computer-based” or “electronic” only if offline products are used. Use “virtual” only in the context of “virtual reality” (3-D worlds). Use “online” only in the context of “online support groups”. Complement or substitute product names with broader terms for the class of products (such as “mobile” or “smart phone” instead of “iphone”), especially if the application runs on different platforms.

1 2 3 4 5

subitem not at all important      essential

**Does your paper address subitem 1a-i? ***

Copy and paste relevant sections from manuscript title (include quotes in quotation marks "like this" to indicate direct quotes from your manuscript), or elaborate on this item by providing additional information not in the ms, or briefly explain why the item is not applicable/relevant for your study

“mobile health interventions”

**1a-ii) Non-web-based components or important co-interventions in title**

Mention non-web-based components or important co-interventions in title, if any (e.g., “with telephone support”).

1 2 3 4 5

subitem not at all important      essential

**Does your paper address subitem 1a-ii?**

Copy and paste relevant sections from manuscript title (include quotes in quotation marks "like this" to indicate direct quotes from your manuscript), or elaborate on this item by providing additional information not in the ms, or briefly explain why the item is not applicable/relevant for your study

Item is not applicable: No further co-interventions are carried out in this study.

**1a-iii) Primary condition or target group in the title**

Mention primary condition or target group in the title, if any (e.g., “for children with Type I Diabetes”) Example: A Web-based and Mobile Intervention with Telephone Support for Children with Type I Diabetes: Randomized Controlled Trial

1 2 3 4 5

subitem not at all important      essential

**Does your paper address subitem 1a-iii? ***

Copy and paste relevant sections from manuscript title (include quotes in quotation marks "like this" to indicate direct quotes from your manuscript), or elaborate on this item by providing additional information not in the ms, or briefly explain why the item is not applicable/relevant for your study

“family setting”

**1b) ABSTRACT: Structured summary of trial design, methods, results, and conclusions**

NPT extension: Description of experimental treatment, comparator, care providers, centers, and blinding status.

**1b-i) Key features/functionalities/components of the intervention and comparator in the METHODS section of the ABSTRACT**

Mention key features/functionalities/components of the intervention and comparator in the abstract. If possible, also mention theories and principles used for designing the site. Keep in mind the needs of systematic reviewers and indexers by including important synonyms. (Note: Only report in the abstract what the main paper is reporting. If this information is missing from the main body of text, consider adding it)

1 2 3 4 5

subitem not at all important      essential

**Does your paper address subitem 1b-i? ***

Copy and paste relevant sections from the manuscript abstract (include quotes in quotation marks "like this" to indicate direct quotes from your manuscript), or elaborate on this item by providing additional information not in the ms, or briefly explain why the item is not applicable/relevant for your study

“A smartphone app based on behavior change theories and -techniques was developed, implemented, and evaluated in a collective family setting with family members individually and cooperatively using the SF-app. Therefore, a cluster-randomized controlled trial including three measurement occasions was conducted. The intervention group (IG) used the app for three consecutive weeks, whereas the control group (CG) received no treatment. Baseline- (T0) and post-measurements (T1) included PA (self-reported and accelerometry) and HE measurement (self-reported fruit and vegetable intake (FVI)) as primary outcomes. Secondary outcomes (self-reported) were intrinsic motivation, behavior-specific self-efficacy, and the Family Health Climate (FHC). Four weeks following T1, a follow-up assessment (T2) was completed by participants, consisting of all questionnaire items to assess the stability of the intervention effects. Multilevel analyses were implemented in R to acknowledge the hierarchical structure of persons (level 1) clustered in families (level 2).”

**1b-ii) Level of human involvement in the METHODS section of the ABSTRACT**

Clarify the level of human involvement in the abstract, e.g., use phrases like “fully automated” vs. “therapist/nurse/care provider/physician-assisted” (mention number and expertise of providers involved, if any). (Note: Only report in the abstract what the main paper is reporting. If this information is missing from the main body of text, consider adding it)

1 2 3 4 5

subitem not at all important      essential

**Does your paper address subitem 1b-ii?**

Copy and paste relevant sections from the manuscript abstract (include quotes in quotation marks "like this" to indicate direct quotes from your manuscript), or elaborate on this item by providing additional information not in the ms, or briefly explain why the item is not applicable/relevant for your study

Not applicable: no human involvement during intervention phase

**1b-iii) Open vs. closed, web-based (self-assessment) vs. face-to-face assessments in the METHODS** **section of the ABSTRACT**

Mention how participants were recruited (online vs. offline), e.g., from an open access website or from a clinic or a closed online user group (closed usergroup trial), and clarify if this was a purely web-based trial, or there were face-to-face components (as part of the intervention or for assessment). Clearly say if outcomes were self-assessed through questionnaires (as common in web-based trials). Note: In traditional offline trials, an open trial (open-label trial) is a type of clinical trial in which both the researchers and participants know which treatment is being administered. To avoid confusion, use “blinded” or “unblinded” to indicated the level of blinding instead of “open”, as “open” in web-based trials usually refers to “open access” (i.e. participants can self-enrol). (Note: Only report in the abstract what the main paper is reporting. If this information is missing from the main body of text, consider adding it)

1 2 3 4 5

subitem not at all important      essential

**Does your paper address subitem 1b-iii?**

Copy and paste relevant sections from the manuscript abstract (include quotes in quotation marks "like this" to indicate direct quotes from your manuscript), or elaborate on this item by providing additional information not in the ms, or briefly explain why the item is not applicable/relevant for your study

Participants were recruited offline, outcomes were self-assessed (questionnaire and accelerometry). Please refer to the abstract section method.

**1b-iv) RESULTS section in abstract must contain use data**

Report number of participants enrolled/assessed in each group, the use/uptake of the intervention (e.g., attrition/adherence metrics, use over time, number of logins etc.), in addition to primary/secondary outcomes. (Note: Only report in the abstract what the main paper is reporting. If this information is missing from the main body of text, consider adding it)

1 2 3 4 5

subitem not at all important      essential

**Does your paper address subitem 1b-iv?**

Copy and paste relevant sections from the manuscript abstract (include quotes in quotation marks "like this" to indicate direct quotes from your manuscript), or elaborate on this item by providing additional information not in the ms, or briefly explain why the item is not applicable/relevant for your study

“Overall, 48 families (22 KG, *n* = 68 participants; 26 IG, *n* = 88) were recruited for the study. Two families (1 KG, *n* = 4; 1 IG, *n* = 4) chose to drop out of the study due to personal reasons before T_0_.”

**1b-v) CONCLUSIONS/DISCUSSION in abstract for negative trials**

Conclusions/Discussions in abstract for negative trials: Discuss the primary outcome - if the trial is negative (primary outcome not changed), and the intervention was not used, discuss whether negative results are attributable to lack of uptake and discuss reasons. (Note: Only report in the abstract what the main paper is reporting. If this information is missing from the main body of text, consider adding it)

1 2 3 4 5

subitem not at all important      essential

**Does your paper address subitem 1b-v?**

Copy and paste relevant sections from the manuscript abstract (include quotes in quotation marks "like this" to indicate direct quotes from your manuscript), or elaborate on this item by providing additional information not in the ms, or briefly explain why the item is not applicable/relevant for your study

“The SMART*FAMILY* trial aimed to assess the effectiveness of a mobile health (mHealth) intervention in promoting physical activity (PA) and healthy eating (HE) in families. Despite incorporating behavior change techniques rooted in family life and psychological theories, the intervention did not yield significant increases in PA and HE levels among the physically active participants. Previous studies on digital health interventions have shown mixed results and family-based mHealth interventions are scarce, with limited focus on whole-family behavior and randomized controlled trials. Enhancing intervention effectiveness may involve incorporating health literacy, just-in-time adaptive interventions, and more advanced features in future app development. Further research is needed to better understand intervention engagement and tailor mHealth interventions to individuals for enhanced effectiveness in primary prevention efforts.”

## INTRODUCTION

**2a) In INTRODUCTION: Scientific background and explanation of rationale**

**2a-i) Problem and the type of system/solution**

Describe the problem and the type of system/solution that is object of the study: intended as stand-alone intervention vs. incorporated in broader health care program? Intended for a particular patient population? Goals of the intervention, e.g., being more cost-effective to other interventions, replace or complement other solutions? (Note: Details about the intervention are provided in “Methods” under 5)

1 2 3 4 5

subitem not at all important      essential

**Does your paper address subitem 2a-i? ***

Copy and paste relevant sections from the manuscript (include quotes in quotation marks "like this" to indicate direct quotes from your manuscript), or elaborate on this item by providing additional information not in the ms, or briefly explain why the item is not applicable/relevant for your study

Please refer to the third and fourth paragraph in the section “ Background” as well as the “Objective” paragraph to get more information: “Therefore, we aimed with the SMART*FAMILY* trial at developing a mobile stand-alone smartphone intervention app that incorporates the family as a social system of high relevance for its single members. We designed and evaluated a multicomponent mHealth intervention that aimed to improve PA and HE in families. The development of the app was based on behavior change theories including self-determination theory and the use of BCTs. The behavior of children *and* parents was targeted to induce family- and individual-based behavior changes. Specifically, family members were using the SMART*FAMILY* app individually and cooperatively. For more details and the complete study protocol see [67]. A positive influence of the mHealth intervention on PA variables steps and MVPA and the HE variable FVI in the whole family was hypothesized.“

**2a-ii) Scientific background, rationale: What is known about the (type of) system**

Scientific background, rationale: What is known about the (type of) system that is the object of the study (be sure to discuss the use of similar systems for other conditions/diagnoses, if appropiate), motivation for the study, i.e. what are the reasons for and what is the context for this specific study, from which stakeholder viewpoint is the study performed, potential impact of findings [2]. Briefly justify the choice of the comparator.

1 2 3 4 5

subitem not at all important      essential

**Does your paper address subitem 2a-ii? ***

Copy and paste relevant sections from the manuscript (include quotes in quotation marks "like this" to indicate direct quotes from your manuscript), or elaborate on this item by providing additional information not in the ms, or briefly explain why the item is not applicable/relevant for your study

See introduction section. The comparator was chosen to detect “real” effects.

**2b) In INTRODUCTION: Specific objectives or hypotheses**

**Does your paper address CONSORT subitem 2b? ***

Copy and paste relevant sections from the manuscript (include quotes in quotation marks "like this" to indicate direct quotes from your manuscript), or elaborate on this item by providing additional information not in the ms, or briefly explain why the item is not applicable/relevant for your study

“Specifically, family members were using the SMART*FAMILY* app individually and cooperatively. For more details and the complete study protocol see [67]. A positive influence of the mHealth intervention on PA variables steps and MVPA and the HE variable FVI in the whole family was hypothesized.”

## METHODS

**3a) Description of trial design (such as parallel, factorial) including allocation ratio**

**Does your paper address CONSORT subitem 3a? ***

Copy and paste relevant sections from the manuscript (include quotes in quotation marks "like this" to indicate direct quotes from your manuscript), or elaborate on this item by providing additional information not in the ms, or briefly explain why the item is not applicable/relevant for your study

“The study was conducted and described according to the corresponding study protocol and the CONSORT-EHEALTH checklist (see Appendix A [68] and is a cluster-randomized controlled trial with two groups: (1) an intervention group receiving the app intervention and (2) a non-intervention control group.The outline of the SF trial is presented in Figure 1. Assessment of outcomes was completed at baseline (T_0_), after the 3-week intervention (or no-intervention) period (post; T_1_), and four weeks after the post-measurement (follow-up; T_2_). As the study protocol is freely accessible (see 67), the study design and measurements will only be described very briefly. For a complete overview, please refer to the study protocol.”

**3b) Important changes to methods after trial commencement (such as eligibility criteria), with** **reasons**

**Does your paper address CONSORT subitem 3b? ***

Copy and paste relevant sections from the manuscript (include quotes in quotation marks "like this" to indicate direct quotes from your manuscript), or elaborate on this item by providing additional information not in the ms, or briefly explain why the item is not applicable/relevant for your study

Assessment of outcomes is completed at baseline (T_0_), after the 3-week intervention (or no-intervention) period (post; T_1_), and four weeks after the post-measurement (follow-up; T_2_). Please refer to the section on study design in Method section.

**3b-i) Bug fixes, Downtimes, Content Changes**

Bug fixes, Downtimes, Content Changes: ehealth systems are often dynamic systems. A description of changes to methods therefore also includes important changes made on the intervention or comparator during the trial (e.g., major bug fixes or changes in the functionality or content) (5-iii) and other “unexpected events” that may have influenced study design such as staff changes, system failures/downtimes, etc. [2].

1 2 3 4 5

subitem not at all important      essential

**Does your paper address subitem 3b-i?**

Copy and paste relevant sections from the manuscript (include quotes in quotation marks "like this" to indicate direct quotes from your manuscript), or elaborate on this item by providing additional information not in the ms, or briefly explain why the item is not applicable/relevant for your study

Please refer to the Study Protocol (section on Development) to get more information.

**4a) Eligibility criteria for participants**

**Does your paper address CONSORT subitem 4a? ***

Copy and paste relevant sections from the manuscript (include quotes in quotation marks "like this" to indicate direct quotes from your manuscript), or elaborate on this item by providing additional information not in the ms, or briefly explain why the item is not applicable/relevant for your study

“Households comprising at least one adult caregiver and one child over ten years old residing together could participate in the research. If applicable, other siblings were welcome to join the project as well. Everyone taking part had to possess basic proficiency in handling mobile devices and communicate effectively in German. However, exceptions might have occurred for younger siblings who met this requirement.”

**4a-i) Computer / Internet literacy**

Computer / Internet literacy is often an implicit “de facto” eligibility criterion - this should be explicitly clarified.

1 2 3 4 5

subitem not at all important      essential

**Does your paper address subitem 4a-i?**

Copy and paste relevant sections from the manuscript (include quotes in quotation marks "like this" to indicate direct quotes from your manuscript), or elaborate on this item by providing additional information not in the ms, or briefly explain why the item is not applicable/relevant for your study

“Everyone taking part had to possess basic proficiency in handling mobile devices and communicate effectively in German”.

**4a-ii) Open vs. closed, web-based vs. face-to-face assessments:**

Open vs. closed, web-based vs. face-to-face assessments: Mention how participants were recruited (online vs. offline), e.g., from an open access website or from a clinic, and clarify if this was a purely web-based trial, or there were face-to-face components (as part of the intervention or for assessment), i.e., to what degree got the study team to know the participant. In online-only trials, clarify if participants were quasi-anonymous and whether having multiple identities was possible or whether technical or logistical measures (e.g., cookies, email confirmation, phone calls) were used to detect/prevent these.

1 2 3 4 5

subitem not at all important      essential

**Does your paper address subitem 4a-ii? ***

Copy and paste relevant sections from the manuscript (include quotes in quotation marks "like this" to indicate direct quotes from your manuscript), or elaborate on this item by providing additional information not in the ms, or briefly explain why the item is not applicable/relevant for your study

“Participants were recruited in schools, school holiday programs, music schools, and sports clubs, via personal communication, newspapers, social media and email distribution lists of the Karlsruhe Institute of Technology. Overall, 48 families (22 KG, *n* = 68 participants; 26 IG, *n* = 88) were recruited for the study. Two families (1 KG, *n* = 4; 1 IG, *n* = 4) chose to drop out of the study due to personal reasons before T_0_.”

**4a-iii) Information giving during recruitment**

Information given during recruitment. Specify how participants were briefed for recruitment and in the informed consent procedures (e.g., publish the informed consent documentation as appendix, see also item X26), as this information may have an effect on user self-selection, user expectation and may also bias results.

1 2 3 4 5

subitem not at all important      essential

**Does your paper address subitem 4a-iii?**

Copy and paste relevant sections from the manuscript (include quotes in quotation marks "like this" to indicate direct quotes from your manuscript), or elaborate on this item by providing additional information not in the ms, or briefly explain why the item is not applicable/relevant for your study

Information can be found in the Study Protocol: “Whereas intervention group participants are told about the mHealth nature of the study, control group participants are only told to take part in an epidemiologic assessment of physical activity levels, making it essential to wear the accelerometers two times for one week and to answer several questions over the course of 10 weeks in order to gain reliable and valid results.”

**4b) Settings and locations where the data were collected**

**Does your paper address CONSORT subitem 4b? ***

Copy and paste relevant sections from the manuscript (include quotes in quotation marks "like this" to indicate direct quotes from your manuscript), or elaborate on this item by providing additional information not in the ms, or briefly explain why the item is not applicable/relevant for your study

Data are collected in family setting. Location of data collection are not limited to home/school/work. This application adapted to all location in everyday life.

**4b-i) Report if outcomes were (self-)assessed through online questionnaires**

Clearly report if outcomes were (self-)assessed through online questionnaires (as common in web-based trials) or otherwise.

1 2 3 4 5

subitem not at all important      essential

**Does your paper address subitem 4b-i? ***

Copy and paste relevant sections from the manuscript (include quotes in quotation marks "like this" to indicate direct quotes from your manuscript), or elaborate on this item by providing additional information not in the ms, or briefly explain why the item is not applicable/relevant for your study

In SF, outcomes were assessed using paper-pencil questionnaires (self-report) and diaries (self-report), as well as device-based through accelerometry.

**4b-ii) Report how institutional affiliations are displayed**

Report how institutional affiliations are displayed to potential participants [on ehealth media], as affiliations with prestigious hospitals or universities may affect volunteer rates, use, and reactions with regards to an intervention. (Not a required item – describe only if this may bias results)

1 2 3 4 5

subitem not at all important      essential

**Does your paper address subitem 4b-ii?**

Copy and paste relevant sections from the manuscript (include quotes in quotation marks "like this" to indicate direct quotes from your manuscript), or elaborate on this item by providing additional information not in the ms, or briefly explain why the item is not applicable/relevant for your study

Institutional affiliations are only mentioned in contact information. No bias is assumed.

**5) The interventions for each group with sufficient details to allow replication, including how and** **when they were actually administered**

**5-i) Mention names, credential, affiliations of the developers, sponsors, and owners**

Mention names, credential, affiliations of the developers, sponsors, and owners [6] (if authors/evaluators are owners or developer of the software, this needs to be declared in a “Conflict of interest” section or mentioned elsewhere in the manuscript).

1 2 3 4 5

subitem not at all important      essential

**Does your paper address subitem 5-i?**

Copy and paste relevant sections from the manuscript (include quotes in quotation marks "like this" to indicate direct quotes from your manuscript), or elaborate on this item by providing additional information not in the ms, or briefly explain why the item is not applicable/relevant for your study

“All authors declare they do not have any conflicts of interest. Authors/evaluators are distinct from the developers/sponsors of the intervention.” Please refer to the section on Development in the Study Protocol to get more information. “Both, SF and SF2.0 were developed as part of the SMARTFAMILY project which is part of consortium project SMARTACT and its toolbox encompassing mobile interventions for promoting PA and HE (see for example [9–12]; www.uni-konstanz.de/smartact). (…).”

**5-ii) Describe the history/development process**

Describe the history/development process of the application and previous formative evaluations (e.g., focus groups, usability testing), as these will have an impact on adoption/use rates and help with interpreting results.

1 2 3 4 5

subitem not at all important      essential

**Does your paper address subitem 5-ii?**

Copy and paste relevant sections from the manuscript (include quotes in quotation marks "like this" to indicate direct quotes from your manuscript), or elaborate on this item by providing additional information not in the ms, or briefly explain why the item is not applicable/relevant for your study

Please refer to the section on Development in the Study Protocol to get more information. “Generating SF and SF2.0 appearance and content are adapted and changed in iterative processes throughout the invention phases and pilot studies, but not throughout a trial. Both apps are preliminary tested for usability and feasibility. (…).”

**5-iii) Revisions and updating**

Revisions and updating. Clearly mention the date and/or version number of the application/intervention (and comparator, if applicable) evaluated, or describe whether the intervention underwent major changes during the evaluation process, or whether the development and/or content was “frozen” during the trial. Describe dynamic components such as news feeds or changing content which may have an impact on the replicability of the intervention (for unexpected events see item 3b).

1 2 3 4 5

subitem not at all important      essential

**Does your paper address subitem 5-iii?**

Copy and paste relevant sections from the manuscript (include quotes in quotation marks "like this" to indicate direct quotes from your manuscript), or elaborate on this item by providing additional information not in the ms, or briefly explain why the item is not applicable/relevant for your study

Please refer to the section on Development in the Study Protocol to get more information. “Generating SF and SF2.0 appearance and content are adapted and changed in iterative processes throughout the invention phases and pilot studies, but not throughout a trial. Both apps are preliminary tested for usability and feasibility (…).”

**5-iv) Quality assurance methods**

Provide information on quality assurance methods to ensure accuracy and quality of information provided [1], if applicable.

1 2 3 4 5

subitem not at all important      essential

**Does your paper address subitem 5-iv?**

Copy and paste relevant sections from the manuscript (include quotes in quotation marks "like this" to indicate direct quotes from your manuscript), or elaborate on this item by providing additional information not in the ms, or briefly explain why the item is not applicable/relevant for your study

To assure quality, validated scales were used to assess outcomes of interest.

**5-v) Ensure replicability by publishing the source code, and/or providing screenshots/screen-capture video, and/or providing flowcharts of the algorithms used**

Ensure replicability by publishing the source code, and/or providing screenshots/screen-capture video, and/or providing flowcharts of the algorithms used. Replicability (i.e., other researchers should in principle be able to replicate the study) is a hallmark of scientific reporting.

1 2 3 4 5

subitem not at all important      essential

**Does your paper address subitem 5-v?**

Copy and paste relevant sections from the manuscript (include quotes in quotation marks "like this" to indicate direct quotes from your manuscript), or elaborate on this item by providing additional information not in the ms, or briefly explain why the item is not applicable/relevant for your study

The trials will be replicable after proposed release of the SMARTACT Toolbox following successful evaluation of all apps and features comprised.

**5-vi) Digital preservation**

Digital preservation: Provide the URL of the application, but as the intervention is likely to change or disappear over the course of the years; also make sure the intervention is archived (Internet Archive, [webcitation.org](https://www.google.com/url?q=http://webcitation.org&sa=D&ust=1588063950866000&usg=AFQjCNFaJgO0fUMaxYw6Vurx5RchV1jfrA), and/or publishing the source code or screenshots/videos alongside the article). As pages behind login screens cannot be archived, consider creating demo pages which are accessible without login.

1 2 3 4 5

subitem not at all important      essential

**Does your paper address subitem 5-vi?**

Copy and paste relevant sections from the manuscript (include quotes in quotation marks "like this" to indicate direct quotes from your manuscript), or elaborate on this item by providing additional information not in the ms, or briefly explain why the item is not applicable/relevant for your study

The final mobile intervention is planned to be made publicly accessible through the SMARTACT Toolbox. Demo pages are shown in the Study Protocol. Please refer to Figures 4 and 5 for further details.

**5-vii) Access**

Access: Describe how participants accessed the application, in what setting/context, if they had to pay (or were paid) or not, whether they had to be a member of specific group. If known, describe how participants obtained “access to the platform and Internet” [1]. To ensure access for editors/reviewers/readers, consider to provide a “backdoor” login account or demo mode for reviewers/readers to explore the application (also important for archiving purposes, see vi).

1 2 3 4 5

subitem not at all important      essential

**Does your paper address subitem 5-vii? ***

Copy and paste relevant sections from the manuscript (include quotes in quotation marks "like this" to indicate direct quotes from your manuscript), or elaborate on this item by providing additional information not in the ms, or briefly explain why the item is not applicable/relevant for your study

“Participants allocated to the IG receive smartphones with the SMARTFAMILY app preinstalled”. App usage was free of charge – participants did not receive and financial compensation in SF.

**5-viii) Mode of delivery, features/functionalities/components of the intervention and comparator,** **and the theoretical framework**

Describe mode of delivery, features/functionalities/components of the intervention and comparator, and the theoretical framework [6] used to design them (instructional strategy [1], behaviour change techniques, persuasive features, etc., see e.g., [7, 8] for terminology). This includes an in-depth description of the content (including where it is coming from and who developed it) [1],” whether [and how] it is tailored to individual circumstances and allows users to track their progress and receive feedback” [6]. This also includes a description of communication delivery channels and – if computer-mediated communication is a component – whether communication was synchronous or asynchronous [6]. It also includes information on presentation strategies [1], including page design principles, average amount of text on pages, presence of hyperlinks to other resources, etc. [1].

1 2 3 4 5

subitem not at all important      essential

**Does your paper address subitem 5-viii? ***

Copy and paste relevant sections from the manuscript (include quotes in quotation marks "like this" to indicate direct quotes from your manuscript), or elaborate on this item by providing additional information not in the ms, or briefly explain why the item is not applicable/relevant for your study

Mode of delivery: Participants in intervention group receive smartphones with the SMARTFAMILY app preinstalled. Participants in control group did not receive any smartphones. “Each smartphone (Samsung Galaxy A5 for SF, Nokia 5 for SF2.0) is connected with an accelerometer (Move 3 (SF) and Move 4 (SF2.0); Movisens GmbH, Karlsruhe, Germany) via Bluetooth Low Energy (BLE).” Please refer to the fourth paragraph on study design and the section on Development within the Study Protocol to get more information about the components. Please refer to Table 1 to see theoretical framework.

**5-ix) Describe use parameters**

Describe use parameters (e.g., intended “doses” and optimal timing for use). Clarify what instructions or recommendations were given to the user, e.g., regarding timing, frequency, heaviness of use, if any, or was the intervention used ad libitum.

1 2 3 4 5

subitem not at all important      essential

**Does your paper address subitem 5-ix?**

Copy and paste relevant sections from the manuscript (include quotes in quotation marks "like this" to indicate direct quotes from your manuscript), or elaborate on this item by providing additional information not in the ms, or briefly explain why the item is not applicable/relevant for your study

There are no instructions given to the participants regarding timing and frequency of use. “The app is designed to be implemented autonomously by participants.” “The only goal-setting instruction given to participants is the recommendation to set collective family goals for the coming week which are slightly higher than the current cumulative performance.” Please refer to the section on study design for further information.

**5-x) Clarify the level of human involvement**

Clarify the level of human involvement (care providers or health professionals, also technical assistance) in the e-intervention or as co-intervention (detail number and expertise of professionals involved, if any, as well as “type of assistance offered, the timing and frequency of the support, how it is initiated, and the medium by which the assistance is delivered”. It may be necessary to distinguish between the level of human involvement required for the trial, and the level of human involvement required for a routine application outside of a RCT setting (discuss under item 21 – generalizability).

1 2 3 4 5

subitem not at all important      essential

**Does your paper address subitem 5-x?**

Copy and paste relevant sections from the manuscript (include quotes in quotation marks "like this" to indicate direct quotes from your manuscript), or elaborate on this item by providing additional information not in the ms, or briefly explain why the item is not applicable/relevant for your study

Item is not applicable. Please see Item 5-ix.

**5-xi) Report any prompts/reminders used**

Report any prompts/reminders used: Clarify if there were prompts (letters, emails, phone calls, SMS) to use the application, what triggered them, frequency etc. It may be necessary to distinguish between the level of prompts/reminders required for the trial, and the level of prompts/reminders for a routine application outside of a RCT setting (discuss under item 21 – generalizability).

1 2 3 4 5

subitem not at all important      essential

**Does your paper address subitem 5-xi? ***

Copy and paste relevant sections from the manuscript (include quotes in quotation marks "like this" to indicate direct quotes from your manuscript), or elaborate on this item by providing additional information not in the ms, or briefly explain why the item is not applicable/relevant for your study

See Study Protocol: “Every evening, at 7PM, participants are asked if they recorded all necessary manual information of physical activity and healthy eating.”

**5-xii) Describe any co-interventions (incl. training/support)**

Describe any co-interventions (incl. training/support): Clearly state any interventions that are provided in addition to the targeted eHealth intervention, as ehealth intervention may not be designed as stand-alone intervention. This includes training sessions and support [1]. It may be necessary to distinguish between the level of training required for the trial, and the level of training for a routine application outside of a RCT setting (discuss under item 21 – generalizability.

1 2 3 4 5

subitem not at all important      essential

**Does your paper address subitem 5-xii? ***

Copy and paste relevant sections from the manuscript (include quotes in quotation marks "like this" to indicate direct quotes from your manuscript), or elaborate on this item by providing additional information not in the ms, or briefly explain why the item is not applicable/relevant for your study

There is no intervention in addition to the app.

**6a) Completely defined pre-specified primary and secondary outcome measures, including how and when they were assessed**

**Does your paper address CONSORT subitem 6a? ***

Copy and paste relevant sections from the manuscript (include quotes in quotation marks "like this" to indicate direct quotes from your manuscript), or elaborate on this item by providing additional information not in the ms, or briefly explain why the item is not applicable/relevant for your study

Please refer to the section on measurements to get completely defined outcome measures. “Primary outcome measures are device-based measured PA (amount of steps taken, minutes of moderate-to-vigorous physical activity) via accelerometers (T0 and T1) as well as self-reported assessed physical activity levels via questionnaire (…).”

**6a-i) Online questionnaires: describe if they were validated for online use and apply CHERRIES items to describe how the questionnaires were designed/deployed**

If outcomes were obtained through online questionnaires, describe if they were validated for online use and apply CHERRIES items to describe how the questionnaires were designed/deployed [9].

1 2 3 4 5

subitem not at all important      essential

**Does your paper address subitem 6a-i?**

Copy and paste relevant sections from manuscript text

“Only validated surveys were used (…). Ethical approval was obtained and data protection ensured. A closed survey design was used and usability and technical functionality of the electronic questionnaire had been tested before fielding the questionnaire.” For reporting of data, the CHERRIES items will be used.

**6a-ii) Describe whether and how “use” (including intensity of use/dosage) was** **defined/measured/monitored**

Describe whether and how “use” (including intensity of use/dosage) was defined/measured/monitored (logins, logfile analysis, etc.). Use/adoption metrics are important process outcomes that should be reported in any ehealth trial.

1 2 3 4 5

subitem not at all important      essential

**Does your paper address subitem 6a-ii?**

Copy and paste relevant sections from manuscript text

In the first trial (SF1.0), no user data was stored and examined.

**6a-iii) Describe whether, how, and when qualitative feedback from participants was obtained**

Describe whether, how, and when qualitative feedback from participants was obtained (e.g., through emails, feedback forms, interviews, focus groups).

1 2 3 4 5

subitem not at all important      essential

**Does your paper address subitem 6a-iii?**

Copy and paste relevant sections from manuscript text

During pilot testing, qualitative feedback from participants was obtained using interviews and questionnaires and responses were integrated in further app development prior to trial onset.

**6b) Any changes to trial outcomes after the trial commenced, with reasons**

**Does your paper address CONSORT subitem 6b? ***

Copy and paste relevant sections from the manuscript (include quotes in quotation marks "like this" to indicate direct quotes from your manuscript), or elaborate on this item by providing additional information not in the ms, or briefly explain why the item is not applicable/relevant for your study

No changes were conducted. “Generating SF and SF2.0 appearance and content are adapted and changed in iterative processes throughout the invention phases and pilot studies, but not throughout a trial.”

**7a) How sample size was determined**

NPT: When applicable, details of whether and how the clustering by care provides or centers was addressed

**7a-i) Describe whether and how expected attrition was taken into account when calculating the** **sample size**

Describe whether and how expected attrition was taken into account when calculating the sample size.

1 2 3 4 5

subitem not at all important      essential

**Does your paper address subitem 7a-i?**

Copy and paste relevant sections from manuscript title (include quotes in quotation marks "like this" to indicate direct quotes from your manuscript), or elaborate on this item by providing additional information not in the ms, or briefly explain why the item is not applicable/relevant for your study

Please refer to the section on Participants within the Study Protocol to get more information. “A priori power analyses using G*Power [13] approved sample size estimation with regard to effect sizes of previous studies on mobile interventions (see [5,14], for example) (small to medium effects; Cohen’s f = 0.25). (…).”

**7b) When applicable, explanation of any interim analyses and stopping guidelines**

**Does your paper address CONSORT subitem 7b? ***

Copy and paste relevant sections from the manuscript (include quotes in quotation marks "like this" to indicate direct quotes from your manuscript), or elaborate on this item by providing additional information not in the ms, or briefly explain why the item is not applicable/relevant for your study

Item not applicable.

**8a) Method used to generate the random allocation sequence**

NPT: When applicable, how care providers were allocated to each trial group

**Does your paper address CONSORT subitem 8a? ***

Copy and paste relevant sections from the manuscript (include quotes in quotation marks "like this" to indicate direct quotes from your manuscript), or elaborate on this item by providing additional information not in the ms, or briefly explain why the item is not applicable/relevant for your study

“Consenting families were randomly assigned to either arm through a straightforward allocation scheme suitable for cluster trials (following 69). While members of the IG were aware of the mHealth aspects of the study, participants in the CG were merely informed about contributing to an epidemiological examination of physical activity and overall health.”

**8b) Type of randomisation; details of any restriction (such as blocking and block size)**

**Does your paper address CONSORT subitem 8b? ***

Copy and paste relevant sections from the manuscript (include quotes in quotation marks "like this" to indicate direct quotes from your manuscript), or elaborate on this item by providing additional information not in the ms, or briefly explain why the item is not applicable/relevant for your study

“Both trials (SF and SF2.0) are cluster-randomized controlled trials with two groups: (1) an intervention group receiving the SF or SF2.0 and (2) a non-intervention control group.”

**9) Mechanism used to implement the random allocation sequence (such as sequentially numbered** **containers), describing any steps taken to conceal the sequence until interventions were assigned**

**Does your paper address CONSORT subitem 9? ***

Copy and paste relevant sections from the manuscript (include quotes in quotation marks "like this" to indicate direct quotes from your manuscript), or elaborate on this item by providing additional information not in the ms, or briefly explain why the item is not applicable/relevant for your study

A random allocation sequence was generated using Microsoft Excel. Only the head of the study knew this sequence. Recruiters were informed about the group of a cluster right before recruitment.

**10) Who generated the random allocation sequence, who enrolled participants, and who assigned** **participants to interventions**

**Does your paper address CONSORT subitem 10? ***

Copy and paste relevant sections from the manuscript (include quotes in quotation marks "like this" to indicate direct quotes from your manuscript), or elaborate on this item by providing additional information not in the ms, or briefly explain why the item is not applicable/relevant for your study

The allocation sequence was generated by the head of the study who also assigned participant (groups) to interventions. Participants were enrolled by employees and / or student assistants.

**11a) If done, who was blinded after assignment to interventions (for example, participants, care** **providers, those assessing outcomes) and how**

NPT: Whether or not administering co-interventions were blinded to group assignment

**11a-i) Specify who was blinded, and who wasn’t**

Specify who was blinded, and who wasn’t. Usually, in web-based trials it is not possible to blind the participants [1, 3] (this should be clearly acknowledged), but it may be possible to blind outcome assessors, those doing data analysis or those administering co-interventions (if any).

1 2 3 4 5

subitem not at all important      essential

**Does your paper address subitem 11a-i? ***

Copy and paste relevant sections from the manuscript (include quotes in quotation marks "like this" to indicate direct quotes from your manuscript), or elaborate on this item by providing additional information not in the ms, or briefly explain why the item is not applicable/relevant for your study

In both trials, participants were blinded and received only group-dependent information about the study design.

**11a-ii) Discuss e.g., whether participants knew which intervention was the “intervention of interest” and which one was the “comparator”**

Informed consent procedures (4a-ii) can create biases and certain expectations - discuss e.g., whether participants knew which intervention was the “intervention of interest” and which one was the “comparator”.

1 2 3 4 5

subitem not at all important      essential

**Does your paper address subitem 11a-ii?**

Copy and paste relevant sections from the manuscript (include quotes in quotation marks "like this" to indicate direct quotes from your manuscript), or elaborate on this item by providing additional information not in the ms, or briefly explain why the item is not applicable/relevant for your study

Participant did not know about “intervention of interest”. “While members of the IG were aware of the mHealth aspects of the study, participants in the CG were merely informed about contributing to an epidemiological examination of physical activity and overall health.”

**11b) If relevant, description of the similarity of interventions**

(this item is usually not relevant for ehealth trials as it refers to similarity of a placebo or sham intervention to a active medication/intervention)

**Does your paper address CONSORT subitem 11b? ***

Copy and paste relevant sections from the manuscript (include quotes in quotation marks "like this" to indicate direct quotes from your manuscript), or elaborate on this item by providing additional information not in the ms, or briefly explain why the item is not applicable/relevant for your study

Item not applicable. There was no second intervention.

**12a) Statistical methods used to compare groups for primary and secondary outcomes**

NPT: When applicable, details of whether and how the clustering by care providers or centers was addressed

**Does your paper address CONSORT subitem 12a? ***

Copy and paste relevant sections from the manuscript (include quotes in quotation marks "like this" to indicate direct quotes from your manuscript), or elaborate on this item by providing additional information not in the ms, or briefly explain why the item is not applicable/relevant for your study

Please refer to the section on Statistical Analysis to get more information about statistical methods. “The analyses were run with different packages of R [84] and RStudio [85]. The package ‘ggplot2’ was used for visualizations [86] following the instructions of Allan and colleagues [87]. Mixed models were calculated using the package ‘lmerTest’ [88] with participants (level 1) nested in families (level 2) to acknowledge the hierarchical structure of the data. The result tables of the regression analyses were generated using the package ‘sjPlot’ [89]. Here, six final models were calculated, one with each measurement method and outcome parameter (1 steps, 3 MVPA, 2 FVI intake per week) as dependent variables. Assumptions were checked using the visualization of the ‘performance’ package [90]. A hierarchical approach was used for the inclusion of the control variables and the model fit was assessed with the Akaike information criterion (AIC) for sensitivity analysis. Additionally, two models were calculated for joint PA and NU based on the family mean values without the random factor family since it was defined as "all family members were present".

The predictor group (i.e. control = 0, intervention = 1) x time (dummy coded with T_0_ as reference for T_1_ and T_2_) was included in the models to evaluate the interaction effect (main effect) of the intervention on the eight outcome parameters. To assess sensitivity regarding the additional variables, the secondary outcome parameters self-efficacy, intrinsic motivation, and the family health climate were added either referring to PA or FVI depending on the outcome and the control variables health status, population (adult = 0, children = 1), sex (0 = male, 1 = female) and non-wear time per week - only for the device-based measured PA models - were tested for the inclusion in the random effect models. Additionally, the inclusion of random slopes and random intercepts were evaluated based on the model fit. The level of statistical significance was set a priori to α < 0.05”

**12a-i) Imputation techniques to deal with attrition / missing values**

Imputation techniques to deal with attrition / missing values: Not all participants will use the intervention/comparator as intended and attrition is typically high in ehealth trials. Specify how participants who did not use the application or dropped out from the trial were treated in the statistical analysis (a complete case analysis is strongly discouraged, and simple imputation techniques such as LOCF may also be problematic [4]).

1 2 3 4 5

subitem not at all important      essential

**Does your paper address subitem 12a-i? ***

Copy and paste relevant sections from the manuscript (include quotes in quotation marks "like this" to indicate direct quotes from your manuscript), or elaborate on this item by providing additional information not in the ms, or briefly explain why the item is not applicable/relevant for your study

Due to the fact that missing data was not completely at random (MCAR), no imputation techniques were applied.

**12b) Methods for additional analyses, such as subgroup analyses and adjusted analyses**

**Does your paper address CONSORT subitem 12b? ***

Copy and paste relevant sections from the manuscript (include quotes in quotation marks "like this" to indicate direct quotes from your manuscript), or elaborate on this item by providing additional information not in the ms, or briefly explain why the item is not applicable/relevant for your study

Due to the nested design, sensitivity analyses were performed and can be found in the Appendix.

**X26) REB/IRB Approval and Ethical Considerations [recommended as subheading under "Methods"] (not a CONSORT item)**

**X26-i) Comment on ethics committee approval**

1 2 3 4 5

subitem not at all important      essential

**Does your paper address subitem X26-i?**

Copy and paste relevant sections from the manuscript (include quotes in quotation marks "like this" to indicate direct quotes from your manuscript), or elaborate on this item by providing additional information not in the ms, or briefly explain why the item is not applicable/relevant for your study

Full ethical approval was obtained from the University of Konstanz (for the consortium SMARTACT) as well as from the Karlsruhe Institute of Technology (for SF and SF2.0). (…) Both trials are conducted in accordance with the Declaration of Helsinki.

**x26-ii) Outline informed consent procedures**

Outline informed consent procedures e.g., if consent was obtained offline or online (how? Checkbox, etc.?), and what information was provided (see 4a-ii). See [6] for some items to be included in informed consent documents.

1 2 3 4 5

subitem not at all important      essential

**Does your paper address subitem X26-ii?**

Copy and paste relevant sections from the manuscript (include quotes in quotation marks "like this" to indicate direct quotes from your manuscript), or elaborate on this item by providing additional information not in the ms, or briefly explain why the item is not applicable/relevant for your study

All participants, children and legal guardians, provided written informed consent prior to commencing the study by signing the informed consent form.

**X26-iii) Safety and security procedures**

Safety and security procedures, incl. privacy considerations, and any steps taken to reduce the likelihood or detection of harm (e.g., education and training, availability of a hotline)

1 2 3 4 5

subitem not at all important      essential

**Does your paper address subitem X26-iii?**

Copy and paste relevant sections from the manuscript (include quotes in quotation marks "like this" to indicate direct quotes from your manuscript), or elaborate on this item by providing additional information not in the ms, or briefly explain why the item is not applicable/relevant for your study

All participant data was pseudonymized. Relevant information was stored on secure servers in accountancy with the data protection policy of the Karlsruhe Institute of Technology. Contact information were provided within the app.

## RESULTS

**13a) For each group, the numbers of participants who were randomly assigned, received intended** **treatment, and were analysed for the primary outcome**

NPT: The number of care providers or centers performing the intervention in each group and the number of patients treated by each care provider in each center

**Does your paper address CONSORT subitem 13a? ***

Copy and paste relevant sections from the manuscript (include quotes in quotation marks "like this" to indicate direct quotes from your manuscript), or elaborate on this item by providing additional information not in the ms, or briefly explain why the item is not applicable/relevant for your study

### Information can be found in the Data availability and participant characteristics section.

**13b) For each group, losses and exclusions after randomisation, together with reasons**

**Does your paper address CONSORT subitem 13b? (NOTE: Preferably, this is shown in a CONSORT flow diagram) ***

Copy and paste relevant sections from the manuscript (include quotes in quotation marks "like this" to indicate direct quotes from your manuscript), or elaborate on this item by providing additional information not in the ms, or briefly explain why the item is not applicable/relevant for your study

Expected losses and exclusions are presented in the Data availability and participant characteristics section

**13b-i) Attrition diagram**

Strongly recommended: An attrition diagram (e.g., proportion of participants still logging in or using the intervention/comparator in each group plotted over time, similar to a survival curve) or other figures or tables demonstrating usage/dose/engagement.

1 2 3 4 5

subitem not at all important      essential

**Does your paper address subitem 13b-i?**

Copy and paste relevant sections from the manuscript or cite the figure number if applicable (include quotes in quotation marks "like this" to indicate direct quotes from your manuscript), or elaborate on this item by providing additional information not in the ms, or briefly explain why the item is not applicable/relevant for your study

Item is not applicable. Item 13 b-i is not applicable as attrition rates were not surveyed within this trial.

**14a) Dates defining the periods of recruitment and follow-up**

**Does your paper address CONSORT subitem 14a? ***

Copy and paste relevant sections from the manuscript (include quotes in quotation marks "like this" to indicate direct quotes from your manuscript), or elaborate on this item by providing additional information not in the ms, or briefly explain why the item is not applicable/relevant for your study

“Testing took place between December 2017 and January 2020.”

**14a-i) Indicate if critical “secular events” fell into the study period**

Indicate if critical “secular events” fell into the study period, e.g., significant changes in Internet resources available or “changes in computer hardware or Internet delivery resources”

1 2 3 4 5

subitem not at all important      essential

**Does your paper address subitem 14a-i?**

Copy and paste relevant sections from the manuscript (include quotes in quotation marks "like this" to indicate direct quotes from your manuscript), or elaborate on this item by providing additional information not in the ms, or briefly explain why the item is not applicable/relevant for your study

No secular events occurred during the study period.

**14b) Why the trial ended or was stopped (early)**

**Does your paper address CONSORT subitem 14b? ***

Copy and paste relevant sections from the manuscript (include quotes in quotation marks "like this" to indicate direct quotes from your manuscript), or elaborate on this item by providing additional information not in the ms, or briefly explain why the item is not applicable/relevant for your study

No. Recruitment was stopped as planned.

**15) A table showing baseline demographic and clinical characteristics for each group**

NPT: When applicable, a description of care providers (case volume, qualification, expertise, etc.) and centers (volume) in each group

**Does your paper address CONSORT subitem 15? ***

Copy and paste relevant sections from the manuscript (include quotes in quotation marks "like this" to indicate direct quotes from your manuscript), or elaborate on this item by providing additional information not in the ms, or briefly explain why the item is not applicable/relevant for your study

Participant characteristics can be found in Table 1.

**15-i) Report demographics associated with digital divide issues**

In ehealth trials it is particularly important to report demographics associated with digital divide issues, such as age, education, gender, social-economic status, computer/Internet/ehealth literacy of the participants, if known.

1 2 3 4 5

subitem not at all important      essential

**Does your paper address subitem 15-i? ***

Copy and paste relevant sections from the manuscript (include quotes in quotation marks "like this" to indicate direct quotes from your manuscript), or elaborate on this item by providing additional information not in the ms, or briefly explain why the item is not applicable/relevant for your study

Table 1 displays relevant parameters, i.e. population, sex, age and BMI.

**16) For each group, number of participants (denominator) included in each analysis and whether the analysis was by original assigned groups**

**16-i) Report multiple “denominators” and provide definitions**

Report multiple “denominators” and provide definitions: Report N’s (and effect sizes) “across a range of study participation [and use] thresholds” [1], e.g., N exposed, N consented, N used more than x times, N used more than y weeks, N participants “used” the intervention/comparator at specific pre-defined time points of interest (in absolute and relative numbers per group). Always clearly define “use” of the intervention.

1 2 3 4 5

subitem not at all important      essential

**Does your paper address subitem 16-i? ***

Copy and paste relevant sections from the manuscript (include quotes in quotation marks "like this" to indicate direct quotes from your manuscript), or elaborate on this item by providing additional information not in the ms, or briefly explain why the item is not applicable/relevant for your study

Inlcuded N’s can be derived from Table 1.

**16-ii) Primary analysis should be intent-to-treat**

Primary analysis should be intent-to-treat, secondary analyses could include comparing only “users”, with the appropriate caveats that this is no longer a randomized sample (see 18-i).

1 2 3 4 5

subitem not at all important      essential

**Does your paper address subitem 16-ii?**

Copy and paste relevant sections from the manuscript (include quotes in quotation marks "like this" to indicate direct quotes from your manuscript), or elaborate on this item by providing additional information not in the ms, or briefly explain why the item is not applicable/relevant for your study

Primary analyses were carried out intent-to-treat.

**17a) For each primary and secondary outcome, results for each group, and the estimated effect size and its precision (such as 95% confidence interval)**

**Does your paper address CONSORT subitem 17a? ***

Copy and paste relevant sections from the manuscript (include quotes in quotation marks "like this" to indicate direct quotes from your manuscript), or elaborate on this item by providing additional information not in the ms, or briefly explain why the item is not applicable/relevant for your study

Yes, all relevant results were reported.

**17a-i) Presentation of process outcomes such as metrics of use and intensity of use**

In addition to primary/secondary (clinical) outcomes, the presentation of process outcomes such as metrics of use and intensity of use (dose, exposure) and their operational definitions is critical. This does not only refer to metrics of attrition (13-b) (often a binary variable), but also to more continuous exposure metrics such as “average session length”. These must be accompanied by a technical description how a metric like a “session” is defined (e.g., timeout after idle time) [1] (report under item 6a).

1 2 3 4 5

subitem not at all important      essential

**Does your paper address subitem 17a-i?**

Copy and paste relevant sections from the manuscript (include quotes in quotation marks "like this" to indicate direct quotes from your manuscript), or elaborate on this item by providing additional information not in the ms, or briefly explain why the item is not applicable/relevant for your study

Unfortunately, data was not stored for this trial. However, usage metrics will be included in the presentation of SF2.0.

**17b) For binary outcomes, presentation of both absolute and relative effect sizes is recommended**

**Does your paper address CONSORT subitem 17b? ***

Copy and paste relevant sections from the manuscript (include quotes in quotation marks "like this" to indicate direct quotes from your manuscript), or elaborate on this item by providing additional information not in the ms, or briefly explain why the item is not applicable/relevant for your study

There are no binary outcomes included.

**18) Results of any other analyses performed, including subgroup analyses and adjusted analyses,** **distinguishing pre-specified from exploratory**

**Does your paper address CONSORT subitem 18? ***

Copy and paste relevant sections from the manuscript (include quotes in quotation marks "like this" to indicate direct quotes from your manuscript), or elaborate on this item by providing additional information not in the ms, or briefly explain why the item is not applicable/relevant for your study

Item is not applicable. All analyses were pre-specified in the Study protocol.

**18-i) Subgroup analysis of comparing only users**

A subgroup analysis of comparing only users is not uncommon in ehealth trials, but if done, it must be stressed that this is a self-selected sample and no longer an unbiased sample from a randomized trial (see 16-iii).

1 2 3 4 5

subitem not at all important      essential

**Does your paper address subitem 18-i?**

Copy and paste relevant sections from the manuscript (include quotes in quotation marks "like this" to indicate direct quotes from your manuscript), or elaborate on this item by providing additional information not in the ms, or briefly explain why the item is not applicable/relevant for your study

Item is not applicable. No subgroup-analyses were performed.

**19) All important harms or unintended effects in each group**

(for specific guidance see CONSORT for harms)

**Does your paper address CONSORT subitem 19? ***

Copy and paste relevant sections from the manuscript (include quotes in quotation marks "like this" to indicate direct quotes from your manuscript), or elaborate on this item by providing additional information not in the ms, or briefly explain why the item is not applicable/relevant for your study

Item is not applicable. There were nor harms or unintended effects.

**19-i) Include privacy breaches, technical problems**

Include privacy breaches, technical problems. This does not only include physical “harm” to participants, but also incidents such as perceived or real privacy breaches [1], technical problems, and other unexpected/unintended incidents. “Unintended effects” also includes unintended positive effects [2].

1 2 3 4 5

subitem not at all important      essential

**Does your paper address subitem 19-i?**

Copy and paste relevant sections from the manuscript (include quotes in quotation marks "like this" to indicate direct quotes from your manuscript), or elaborate on this item by providing additional information not in the ms, or briefly explain why the item is not applicable/relevant for your study

Item is not applicable. There were no privacy breaches or technical problems to deal with.

**19-ii) Include qualitative feedback from participants or observations from staff/researchers**

Include qualitative feedback from participants or observations from staff/researchers, if available, on strengths and shortcomings of the application, especially if they point to unintended/unexpected effects or uses. This includes (if available) reasons for why people did or did not use the application as intended by the developers.

1 2 3 4 5

subitem not at all important      essential

**Does your paper address subitem 19-ii?**

Copy and paste relevant sections from the manuscript (include quotes in quotation marks "like this" to indicate direct quotes from your manuscript), or elaborate on this item by providing additional information not in the ms, or briefly explain why the item is not applicable/relevant for your study

Feedback from participants was obtained in the piloting phase before start of the trial and was incorporated to the final version of the app.

## DISCUSSION

**22) Interpretation consistent with results, balancing benefits and harms, and considering other** **relevant evidence**

NPT: In addition, take into account the choice of the comparator, lack of or partial blinding, and unequal expertise of care providers or centers in each group

**22-i) Restate study questions and summarize the answers suggested by the data, starting with** **primary outcomes and process outcomes (use)**

Restate study questions and summarize the answers suggested by the data, starting with primary outcomes and process outcomes (use).

1 2 3 4 5

subitem not at all important      essential

**Does your paper address subitem 22-i? ***

Copy and paste relevant sections from the manuscript (include quotes in quotation marks "like this" to indicate direct quotes from your manuscript), or elaborate on this item by providing additional information not in the ms, or briefly explain why the item is not applicable/relevant for your study

See “principal Findings”: “The SMART*FAMILY* trial evaluated the effectiveness of a mHealth intervention to increase PA and HE in families. Extending previous research, the behavior of children *and* parents was targeted to induce individual behavior changes that are anchored in daily family life. Moreover, besides a theoretical foundation, several BCTs were included which contribute to the fulfillment of basic psychological needs according to the self-determination theory [62,63]. Overall, no evidence for meaningful and statistically significant increases in PA and HE levels of the intervention was observed in our physically active sample. Guidelines for weekly MVPA were highly overexceeded in the current sample. Although, PA levels were sustained and did not decrease, independent of group affiliation. Intervention effectiveness might be enhanced by adding gamification features, health literacy, a just-in-time adaptive intervention, and more sophisticated app features in further development of the SMART*FAMILY* app.”

**22-ii) Highlight unanswered new questions, suggest future research**

Highlight unanswered new questions, suggest future research.

1 2 3 4 5

subitem not at all important      essential

**Does your paper address subitem 22-ii?**

Copy and paste relevant sections from the manuscript (include quotes in quotation marks "like this" to indicate direct quotes from your manuscript), or elaborate on this item by providing additional information not in the ms, or briefly explain why the item is not applicable/relevant for your study

See “Conclusions”: “Taken together, the evaluation of the SMART*FAMILY* trial expands the existing body of evidence as it investigated the influence of a theory-based mHealth intervention targeting PA and HE in a collective family-based setting. Yet, no evidence for the effectiveness of the trial has been found. This finding, however, might be attributable to an initially active and lean sample. Future evaluations of interventions should therefore also consider (1) methods that go beyond pre-post-follow-up designs to account for the timeliness and complexity of mHealth interventions, (2) recruiting participants of all activity- and weight levels, and (3) control for or restrict ages of children and parents.”

**20) Trial limitations, addressing sources of potential bias, imprecision, and, if relevant, multiplicity of analyses**

**20-i) Typical limitations in ehealth trials**

Typical limitations in ehealth trials: Participants in ehealth trials are rarely blinded. Ehealth trials often look at a multiplicity of outcomes, increasing risk for a Type I error. Discuss biases due to non-use of the intervention/usability issues, biases through informed consent procedures, unexpected events.

1 2 3 4 5

subitem not at all important      essential

**Does your paper address subitem 20-i? ***

Copy and paste relevant sections from the manuscript (include quotes in quotation marks "like this" to indicate direct quotes from your manuscript), or elaborate on this item by providing additional information not in the ms, or briefly explain why the item is not applicable/relevant for your study

See “Strengths and Limitations”.

**21) Generalisability (external validity, applicability) of the trial findings**

NPT: External validity of the trial findings according to the intervention, comparators, patients, and care providers or centers involved in the trial

**21-i) Generalizability to other populations**

Generalizability to other populations: In particular, discuss generalizability to a general Internet population, outside of a RCT setting, and general patient population, including applicability of the study results for other organizations

1 2 3 4 5

subitem not at all important      essential

**Does your paper address subitem 21-i?**

Copy and paste relevant sections from the manuscript (include quotes in quotation marks "like this" to indicate direct quotes from your manuscript), or elaborate on this item by providing additional information not in the ms, or briefly explain why the item is not applicable/relevant for your study

Due to our very active sample, results can not be generalized.

**21-ii) Discuss if there were elements in the RCT that would be different in a routine application** **setting**

Discuss if there were elements in the RCT that would be different in a routine application setting (e.g., prompts/reminders, more human involvement, training sessions or other co-interventions) and what impact the omission of these elements could have on use, adoption, or outcomes if the intervention is applied outside of a RCT setting.

1 2 3 4 5

subitem not at all important      essential

**Does your paper address subitem 21-ii?**

Copy and paste relevant sections from the manuscript (include quotes in quotation marks "like this" to indicate direct quotes from your manuscript), or elaborate on this item by providing additional information not in the ms, or briefly explain why the item is not applicable/relevant for your study

Elements would not differ in a routine setting.

## OTHER INFORMATION

**23) Registration number and name of trial registry**

**Does your paper address CONSORT subitem 23? ***

Copy and paste relevant sections from the manuscript (include quotes in quotation marks "like this" to indicate direct quotes from your manuscript), or elaborate on this item by providing additional information not in the ms, or briefly explain why the item is not applicable/relevant for your study

“The study is registered with the German Clinical Trials Register under the registration number DRKS00010415. The study is funded by the German Federal Ministry of Education and Research.”

**24) Where the full trial protocol can be accessed, if available**

**Does your paper address CONSORT subitem 24? ***

Cite a Multimedia Appendix, other reference, or copy and paste relevant sections from the manuscript (include quotes in quotation marks "like this" to indicate direct quotes from your manuscript), or elaborate on this item by providing additional information not in the ms, or briefly explain why the item is not applicable/relevant for your study

The full protocol can be found here: Wunsch K, Eckert T, Fiedler J, Cleven L, Niermann C, Reiterer H, Renner B, Woll A. Effects of a Collective Family-Based Mobile Health Intervention Called “SMARTFAMILY” on Promoting Physical Activity and Healthy Eating: Protocol for a Randomized Controlled Trial. JMIR Res Protoc 2020;9(11):e20534. doi: [10.2196/20534](https://doi.org/10.2196/20534)

**25) Sources of funding and other support (such as supply of drugs), role of funders**

**Does your paper address CONSORT subitem 25? ***

Copy and paste relevant sections from the manuscript (include quotes in quotation marks "like this" to indicate direct quotes from your manuscript), or elaborate on this item by providing additional information not in the ms, or briefly explain why the item is not applicable/relevant for your study

“This research was supported by the Federal Ministry of Education and Research within the project SMARTACT BMBF Grant (FKZ 01EL1820C).”

**X27) Conflicts of Interest (not a CONSORT item)**

**X27-i) State the relation of the study team towards the system being evaluated**

In addition to the usual declaration of interests (financial or otherwise), also state the relation of the study team towards the system being evaluated, i.e., state if the authors/evaluators are distinct from or identical with the developers/sponsors of the intervention.

1 2 3 4 5

subitem not at all important      essential

**Does your paper address subitem X27-i?**

Copy and paste relevant sections from the manuscript (include quotes in quotation marks "like this" to indicate direct quotes from your manuscript), or elaborate on this item by providing additional information not in the ms, or briefly explain why the item is not applicable/relevant for your study

“All authors declare they do not have any conflicts of interest. Authors/evaluators are distinct from the developers/sponsors of the intervention.” Please see in the method section paragraph “Development” of the Study Protocol for further information how the study team was involved.

**About the CONSORT EHEALTH checklist**

**As a result of using this checklist, did you make changes in your manuscript? ***

yes, major changes

yes, minor changes

no

**What were the most important changes you made as a result of using this checklist?**

**How much time did you spend on going through the checklist INCLUDING making changes in your** **manuscript ***

6 h

**As a result of using this checklist, do you think your manuscript has improved? ***

yes

no

Others:

**Would you like to become involved in the CONSORT EHEALTH group?**

This would involve for example becoming involved in participating in a workshop and writing an "Explanation and Elaboration" document

yes

no

Others:

**Any other comments or questions on CONSORT EHEALTH**

References

1. Ryan RM, Deci EL. Self-determination theory and the facilitation of intrinsic motivation, social development, and well-being. American Psychologist 2000;55(1):68-78. doi:10.1037/0003-066X.55.1.68

2. Deci EL, Ryan RM. Self-Determination Theory. In: van Lange PAM, editor. Handbook of theories of social psychology. Los Angeles, Calif.: Sage; (2012). ISBN:9780857029607. p. 416–437.

3. Whittaker R. Key issues in mobile health and implications for New Zealand. Health Care Inform Rev Onlin 2012;16(2):2-7.

4. König LM, Sproesser G, Schupp HT, Renner B. Describing the Process of Adopting Nutrition and Fitness Apps: Behavior Stage Model Approach. JMIR Mhealth Uhealth 2018;6(3):e55. PMID:29535078

5. Coughlin SS, Whitehead M, Sheats JQ, Mastromonico J, Smith S. A Review of Smartphone Applications for Promoting Physical Activity. Jacobs J Community Med 2016;2(1). PMID:27034992

6. Conroy DE, Yang C-H, Maher JP. Behavior change techniques in top-ranked mobile apps for physical activity. Am J Prev Med 2014;46(6):649-652. PMID:24842742

7. Villinger K, Wahl DR, Boeing H, Schupp HT, Renner B. The effectiveness of app-based mobile interventions on nutrition behaviours and nutrition-related health outcomes: A systematic review and meta-analysis. Obes Rev 2019;20(10):1465-1484. PMID:31353783

8. Iribarren SJ, Cato K, Falzon L, Stone PW. What is the economic evidence for mHealth? A systematic review of economic evaluations of mHealth solutions. PLoS ONE 2017;12(2):e0170581. PMID:28152012

9. Butscher S, Wang Y, Ziesemer K, Wahl, D., König, L., Sproesser G, Renner B, Schupp H, Reiterer H. Lightweight visual data analysis on mobile devices: Providing self-monitoring feedback. In: Cabitza F, Fogli D, Giacomin M, Locoro A, editors. VVH 2016 : Valuable Visualization of Healthcare Information: from the quantified self data to conversations ; Proceedings of the Workshop on Valuable Visualization of Healthcare Information: from the quantified self data to conversations; 2016.

10. Wahl DR, Villinger K, Sproesser G, Schupp HT, Renner B. The behavioral signature of snacking: a visual analysis. The European Health Psychologist 2017;19(5):355-357.

11. Wahl DR, Villinger K, Blumenschein M, König LM, Ziesemer K, Sproesser G, Schupp HT, Renner B. Why We Eat What We Eat: Assessing Dispositional and In-the-Moment Eating Motives by Using Ecological Momentary Assessment. JMIR Mhealth Uhealth 2020;8(1):e13191. PMID:31909719

12. Wunsch K, Gnam J-P, Renner B, Woll A. A family-based m-health intervention to promote physical activity and healthy eating. In: European Health Psycholog Society & Universtiy of Galway, editor. 32nd Conference of the EHPS: health psychology across the lifespan: Uniting Research, Practice and Policy: Conference Abstracts; 2018. p. 412.

13. Faul F, Erdfelder E, Lang A-G, Buchner A. G* Power 3: A flexible statistical power analysis program for the social, behavioral, and biomedical sciences. Behavior research methods 2007;39(2):175-191.

14. Schoeppe S, Alley S, van Lippevelde W, Bray NA, Williams SL, Duncan MJ, Vandelanotte C. Efficacy of interventions that use apps to improve diet, physical activity and sedentary behaviour: a systematic review. Int J Behav Nutr Phys Act 2016;13(1):127. PMID:27927218

15. Esserman D, Allore HG, Travison TG. The Method of Randomization for Cluster-Randomized Trials: Challenges of Including Patients with Multiple Chronic Conditions. Int J Stat Med Res 2016;5(1):2-7. PMID:27478520
