# Supplementary material for: An mHealth Intervention Promoting Physical Activity and Healthy Eating in a Family Setting (SMARTFAMILY): Randomized Controlled Trial
Source: JMIR Mhealth Uhealth. 2024 Apr 26;12:e51201. doi: 10.2196/51201 (PMC11087865; doi:10.2196/51201)
Supplement: Multimedia Appendix 2 [file mhealth_v12i1e51201_app2.docx]

**Table S1**

*Multilevel model analysis for the influence of the three week intervention period on self-reported physical activity (Sixty-Minute Screening Measure) in children. Displayed are the results of the group (control = 0, intervention = 1) x time (dummy coded with T_0_ as reference for T_1_ and T_2_) interaction. All results are displayed using the raw estimates (days with >60 minutes moderate to vigorous physical activity (MVPA)), the standardized Beta (β), 95% confidence intervals (CI), and standardized (std.) 95% CI. Additionally, the within-person variance (σ^2^), the between-person variance (τ_00_* _family_*), the intraclass correlation coefficient (ICC), the number of families (N _family_), the number of observations, and the marginal and conditional R² are displayed.*

|  | **Sixty-Minute Screening Measure**  **days >60 min MVPA/week** | | | | |
| --- | --- | --- | --- | --- | --- |
| *Predictors* | *Estimates* | *β* | *CI* | *std. CI* | *p* |
| (Intercept) | 4.53 | 0.18 | 3.82 – 5.24 | -0.10 – 0.45 | **<0.001** |
| group | -0.28 | -0.08 | -1.24 – 0.68 | -0.36 – 0.20 | 0.568 |
| timepoint [T1] | -0.53 | -0.23 | -1.28 – 0.23 | -0.53 – 0.07 | 0.171 |
| timepoint [T2] | -0.42 | -0.35 | -1.24 – 0.41 | -0.65 – -0.04 | 0.318 |
| group × timepoint [T1] | 0.22 | 0.06 | -0.81 – 1.26 | -0.23 – 0.36 | 0.668 |
| group × timepoint [T2] | -0.33 | -0.09 | -1.41 – 0.76 | -0.40 – 0.22 | 0.550 |
| **Random Effects** | | | | | |
| σ^2^ | 1.91 | | | | |
| τ_00_ _family_ | 1.19 | | | | |
| ICC | 0.38 | | | | |
| N _family_ | 44 | | | | |
| Observations | 166 | | | | |
| Marginal R^2^ / Conditional R^2^ | 0.032 / 0.404 | | | | |

**Table S2**

*Multilevel model analysis for the influence of the three week intervention period on self-reported physical activity (International Physical Activity Questionnaire) in adults. Displayed are the results of the group (control = 0, intervention = 1) x time (dummy coded with T_0_ as reference for T_1_ and T_2_) interaction. All results are displayed using the raw estimates (minutes of moderate to vigorous physical activity (MVPA) per week), the standardized Beta (β), 95% confidence intervals (CI), and standardized (std.) 95% CI. Additionally, the within-person variance (σ^2^), the between-person variance (τ_00_* _family_*), the intraclass correlation coefficient (ICC), the number of families (N _family_), the number of observations, and the marginal and conditional R² are displayed.*

|  | **International Physical Activity Questionnaire**  **MVPA/week** | | | | |
| --- | --- | --- | --- | --- | --- |
| *Predictors* | *Estimates* | *β* | *CI* | *std. CI* | *p* |
| (Intercept) | 966.92 | 0.10 | 580.32 – 1353.51 | -0.20 – 0.40 | **<0.001** |
| group | 162.39 | 0.09 | -367.52 – 692.30 | -0.21 – 0.39 | 0.546 |
| timepoint [T1] | -14.99 | -0.00 | -410.64 – 380.65 | -0.31 – 0.30 | 0.940 |
| timepoint [T2] | -161.00 | -0.18 | -546.21 – 224.20 | -0.48 – 0.12 | 0.410 |
| group × timepoint [T1] | 24.20 | 0.01 | -516.14 – 564.54 | -0.29 – 0.32 | 0.930 |
| group × timepoint [T2] | 7.88 | 0.00 | -516.74 – 532.50 | -0.29 – 0.30 | 0.976 |
| **Random Effects** | | | | | |
| σ^2^ | 410493.17 | | | | |
| τ_00_ _family_ | 425617.62 | | | | |
| ICC | 0.51 | | | | |
| N _family_ | 42 | | | | |
| Observations | 143 | | | | |
| Marginal R^2^ / Conditional R^2^ | 0.015 / 0.516 | | | | |

**Table S3**

*Multilevel model analysis for the influence of the three week intervention period on device-based measured physical activity (accelerometry using 10 second epochs) in adults and children. Displayed are the results of the group (control = 0, intervention = 1) x time (dummy coded with T_0_ as reference for T_1_) interaction. All results are displayed using the raw estimates (minutes of moderate to vigorous physical activity (MVPA) per week), the standardized Beta (β), 95% confidence intervals (CI), and standardized (std.) 95% CI. Additionally, the within-person variance (σ^2^), the between-person variance (τ_00_* _family_*), the intraclass correlation coefficient (ICC), the number of families (N _family_), the number of observations, and the marginal and conditional R² are displayed.*

|  | **Accelerometry**  **MVPA/week** | | | | |
| --- | --- | --- | --- | --- | --- |
| *Predictors* | *Estimates* | *β* | *CI* | *std. CI* | *p* |
| (Intercept) | 693.68 | 0.06 | 604.19 – 783.17 | -0.14 – 0.27 | **<0.001** |
| group | 35.46 | 0.06 | -83.60 – 154.52 | -0.14 – 0.26 | 0.558 |
| timepoint [T1] | -50.88 | -0.16 | -151.95 – 50.19 | -0.41 – 0.08 | 0.322 |
| group × timepoint [T1] | 5.00 | 0.01 | -137.45 – 147.46 | -0.23 – 0.25 | 0.945 |
| **Random Effects** | | | | | |
| σ^2^ | 71334.44 | | | | |
| τ_00_ _family_ | 16994.50 | | | | |
| ICC | 0.19 | | | | |
| N _family_ | 46 | | | | |
| Observations | 240 | | | | |
| Marginal R^2^ / Conditional R^2^ | 0.011 / 0.202 | | | | |

**Table S4**

*Multilevel model analysis for the influence of the three week intervention period on device-based measured physical activity (accelerometry using 10 second epochs) in adults and children. Displayed are the results of the group (control = 0, intervention = 1) x time (dummy coded with T_0_ as reference for T_1_) interaction. All results are displayed using the raw estimates (step count per week), the standardized Beta (β), 95% confidence intervals (CI), and standardized (std.) 95% CI. Additionally, the within-person variance (σ^2^), the between-person variance (τ_00_* _family_*), the intraclass correlation coefficient (ICC), the number of families (N _family_), the number of observations, and the marginal and conditional R² are displayed.*

|  | **Accelerometry**  **step count/week** | | | | |
| --- | --- | --- | --- | --- | --- |
| *Predictors* | *Estimates* | *β* | *CI* | *std. CI* | *p* |
| (Intercept) | 60850.67 | 0.03 | 53094.75 – 68606.59 | -0.17 – 0.24 | **<0.001** |
| group | 6282.35 | 0.13 | -4057.62 – 16622.33 | -0.08 – 0.33 | 0.233 |
| timepoint [T1] | -3820.04 | -0.09 | -12084.75 – 4444.67 | -0.32 – 0.15 | 0.363 |
| group × timepoint [T1] | 3142.66 | 0.06 | -8525.13 – 14810.45 | -0.17 – 0.30 | 0.596 |
| **Random Effects** | | | | | |
| σ^2^ | 474693821.16 | | | | |
| τ_00_ _family_ | 149342927.96 | | | | |
| ICC | 0.24 | | | | |
| N _family_ | 46 | | | | |
| Observations | 240 | | | | |
| Marginal R^2^ / Conditional R^2^ | 0.027 / 0.260 | | | | |

**Table S5**

*Multilevel model analysis for the influence of the three week intervention period on self-reported fruit and vegetable intake (single item questionnaire) in adults and children. Displayed are the results of the group (control = 0, intervention = 1) x time (dummy coded with T_0_ as reference for T_1_ and T_2_) interaction. All results are displayed using the raw estimates (fruit and vegetable portions per week), the standardized Beta (β), 95% confidence intervals (CI), and standardized (std.) 95% CI. Additionally, the within-person variance (σ^2^), the between-person variance (τ_00_* _family_*), the intraclass correlation coefficient (ICC), the number of families (N _family_), the number of observations, and the marginal and conditional R² are displayed.*

|  | **Questionnaire**  **fruit and vegetable intake/week** | | | | |
| --- | --- | --- | --- | --- | --- |
| *Predictors* | *Estimates* | *β* | *CI* | *std. CI* | *p* |
| (Intercept) | 11.17 | -0.01 | 7.88 – 14.46 | -0.24 – 0.22 | **<0.001** |
| group | 4.53 | 0.23 | 0.02 – 9.05 | 0.00 – 0.46 | **0.049** |
| timepoint [T1] | 0.09 | 0.03 | -2.68 – 2.85 | -0.15 – 0.22 | 0.951 |
| timepoint [T2] | 0.31 | 0.01 | -2.46 – 3.07 | -0.18 – 0.20 | 0.828 |
| group × timepoint [T1] | 0.44 | 0.02 | -3.24 – 4.12 | -0.17 – 0.21 | 0.815 |
| group × timepoint [T2] | -0.35 | -0.02 | -4.08 – 3.37 | -0.21 – 0.17 | 0.852 |
| **Random Effects** | | | | | |
| σ^2^ | 53.91 | | | | |
| τ_00_ _family_ | 39.20 | | | | |
| ICC | 0.42 | | | | |
| N _family_ | 44 | | | | |
| Observations | 375 | | | | |
| Marginal R^2^ / Conditional R^2^ | 0.053 / 0.452 | | | | |

**Table S6**

*Multilevel model analysis for the influence of the three week intervention period on self-reported fruit and vegetable intake (diary) in adults and children. Displayed are the results of the group (control = 0, intervention = 1) x time (dummy coded with T_0_ as reference for T_1_) interaction. All results are displayed using the raw estimates (fruit and vegetable portions per week), the standardized Beta (β), 95% confidence intervals (CI), and standardized (std.) 95% CI. Additionally, the within-person variance (σ^2^), the between-person variance (τ_00_* _family_*), the intraclass correlation coefficient (ICC), the number of families (N _family_), the number of observations, and the marginal and conditional R² are displayed.*

|  | **Diary**  **fruit and vegetable intake/week** | | | | |
| --- | --- | --- | --- | --- | --- |
| *Predictors* | *Estimates* | *β* | *CI* | *std. CI* | *p* |
| (Intercept) | 13.46 | -0.03 | 10.14 – 16.79 | -0.26 – 0.20 | **<0.001** |
| group | 3.34 | 0.17 | -1.28 – 7.96 | -0.07 – 0.40 | 0.156 |
| timepoint [T1] | -0.18 | 0.08 | -2.84 – 2.47 | -0.11 – 0.27 | 0.892 |
| group × timepoint [T1] | 1.85 | 0.09 | -1.87 – 5.57 | -0.09 – 0.28 | 0.328 |
| **Random Effects** | | | | | |
| σ^2^ | 55.27 | | | | |
| τ_00_ _family_ | 40.82 | | | | |
| ICC | 0.42 | | | | |
| N _family_ | 43 | | | | |
| Observations | 258 | | | | |
| Marginal R^2^ / Conditional R^2^ | 0.048 / 0.452 | | | | |

**Table S7**

*Linear model analysis for the influence of the three week intervention period on self-reported common physical activities (questionnaire) in the families. Displayed are the results of the group (control = 0, intervention = 1) x time (dummy coded with T_0_ as reference for T_1_) interaction. All results are displayed using the raw estimates (joint physical activities per week), the standardized Beta (β), 95% confidence intervals (CI), and standardized (std.) 95% CI. Additionally, the number of families (N _family_), the number of observations, and the R² and adjusted R² are displayed.*

|  | **Questionnaire**  **joint physical activities/week** | | | | |
| --- | --- | --- | --- | --- | --- |
| *Predictors* | *Estimates* | *β* | *CI* | *std. CI* | *p* |
| (Intercept) | 0.51 | -0.00 | 0.36 – 0.67 | -0.12 – 0.11 | **<0.001** |
| group | 0.45 | 0.25 | 0.25 – 0.66 | 0.14 – 0.36 | **<0.001** |
| timepoint [T1] | 0.15 | -0.01 | -0.07 – 0.36 | -0.17 – 0.14 | 0.178 |
| timepoint [T2] | 0.08 | 0.02 | -0.14 – 0.30 | -0.13 – 0.18 | 0.478 |
| group × timepoint [T1] | -0.28 | -0.15 | -0.56 – 0.01 | -0.31 – 0.00 | 0.055 |
| group × timepoint [T2] | -0.10 | -0.05 | -0.38 – 0.19 | -0.21 – 0.10 | 0.502 |
| Observations | 933 | | | | |
| R^2^ / R^2^ adjusted | 0.036 / 0.031 | | | | |

**Table S8**

*Linear model analysis for the influence of the three week intervention period on self-reported common meals (questionnaire) in the families. Displayed are the results of the group (control = 0, intervention = 1) x time (dummy coded with T_0_ as reference for T_1_) interaction. All results are displayed using the raw estimates (joint meals per week), the standardized Beta (β), 95% confidence intervals (CI), and standardized (std.) 95% CI. Additionally, the number of families (N _family_), the number of observations, and the R² and adjusted R² are displayed.*

|  | **Questionnaire**  **joint meals/week** | | | | | |
| --- | --- | --- | --- | --- | --- | --- |
| *Predictors* | *Estimates* | *β* | *CI* | *std. CI* | *p* | *std. p* |
| (Intercept) | 8.57 | 0.13 | 7.95 – 9.18 | 0.03 – 0.23 | **<0.001** | **0.010** |
| group | -0.32 | -0.04 | -1.12 – 0.48 | -0.14 – 0.06 | 0.436 | 0.436 |
| timepoint [T1] | -1.25 | -0.31 | -2.13 – -0.38 | -0.45 – -0.17 | **0.005** | **<0.001** |
| timepoint [T2] | -1.03 | -0.08 | -1.90 – -0.15 | -0.23 – 0.07 | **0.022** | 0.275 |
| group × timepoint [T1] | 0.04 | 0.00 | -1.12 – 1.19 | -0.14 – 0.15 | 0.952 | 0.952 |
| group × timepoint [T2] | 1.24 | 0.16 | 0.07 – 2.42 | 0.01 – 0.30 | **0.038** | **0.038** |
| Observations | 1056 | | | | | |
| R^2^ / R^2^ adjusted | 0.023 / 0.018 | | | | | |
