# Supplementary material for: An mHealth Intervention Promoting Physical Activity and Healthy Eating in a Family Setting (SMARTFAMILY): Randomized Controlled Trial
Source: JMIR Mhealth Uhealth. 2024 Apr 26;12:e51201. doi: 10.2196/51201 (PMC11087865; doi:10.2196/51201)
Supplement: Multimedia Appendix 3 [file mhealth_v12i1e51201_app3.docx]

**Table S1**

*Multilevel model analysis for the influence of the three week intervention period on self-reported physical activity (Sixty-Minute Screening Measure) in children. Displayed are the results of the group (control = 0, intervention = 1) x time (dummy coded with T_0_ as reference for T_1_ and T_2_) interaction, and the secondary outcomes self-efficacy, intrinsic motivation, the family health climate (all relating to physical activity), and health status as control variables. All results are displayed using the raw estimates (days with >60 minutes moderate to vigorous physical activity (MVPA)), the standardized Beta (β), 95% confidence intervals (CI), and standardized (std.) 95% CI. Additionally, the within-person variance (σ^2^), the between-person variance (τ_00_* _family_*), the intraclass correlation coefficient (ICC), the number of families (N _family_), the number of observations, and the marginal and conditional R² are displayed.*

|  | **Sixty-Minute Screening Measure**  **days >60 min MVPA/week** | | | | |
| --- | --- | --- | --- | --- | --- |
| *Predictors* | *Estimates* | *β* | *CI* | *std. CI* | *p* |
| (Intercept) | 2.37 | 0.15 | 0.20 – 4.54 | -0.11 – 0.41 | **0.032** |
| group | -0.24 | -0.07 | -1.18 – 0.69 | -0.34 – 0.20 | 0.608 |
| timepoint [T1] | -0.36 | -0.20 | -1.15 – 0.43 | -0.51 – 0.11 | 0.366 |
| timepoint [T2] | -0.18 | -0.24 | -1.08 – 0.71 | -0.57 – 0.08 | 0.686 |
| health | 0.09 | 0.03 | -0.30 – 0.47 | -0.12 – 0.18 | 0.663 |
| self-efficacy PA | 0.02 | 0.08 | -0.02 – 0.05 | -0.10 – 0.27 | 0.379 |
| M intrinsic PA | 0.03 | 0.23 | 0.00 – 0.05 | 0.04 – 0.41 | **0.018** |
| family health climate PA | -0.01 | -0.03 | -0.05 – 0.04 | -0.21 – 0.15 | 0.776 |
| group × timepoint [T1] | 0.02 | 0.01 | -1.07 – 1.12 | -0.30 – 0.32 | 0.966 |
| group × timepoint [T2] | -0.43 | -0.12 | -1.59 – 0.73 | -0.45 – 0.21 | 0.464 |
| **Random Effects** | | | | | |
| σ^2^ | 1.96 | | | | |
| τ_00_ _family_ | 0.85 | | | | |
| ICC | 0.30 | | | | |
| N _family_ | 44 | | | | |
| Observations | 156 | | | | |
| Marginal R^2^ / Conditional R^2^ | 0.101 / 0.373 | | | | |

**Table S2**

*Multilevel model analysis for the influence of the three week intervention period on self-reported physical activity (International Physical Activity Questionnaire) in adults. Displayed are the results of the group (control = 0, intervention = 1) x time (dummy coded with T_0_ as reference for T_1_ and T_2_) interaction, and the secondary outcomes self-efficacy, intrinsic motivation, the family health climate (all relating to physical activity), and health status as control variables. Additionally, sex (0 = male, 1 = female) is included as a control variable. All results are displayed using the raw estimates (minutes of moderate to vigorous physical activity (MVPA) per week), the standardized Beta (β), 95% confidence intervals (CI), and standardized (std.) 95% CI. Additionally, the within-person variance (σ^2^), the between-person variance (τ_00_* _family_*), the intraclass correlation coefficient (ICC), the number of families (N _family_), the number of observations, and the marginal and conditional R² are displayed.*

|  | **International Physical Activity Questionnaire**  **MVPA/week** | | | | |
| --- | --- | --- | --- | --- | --- |
| *Predictors* | *Estimates* | *β* | *CI* | *std. CI* | *p* |
| (Intercept) | 478.56 | 0.06 | -521.17 – 1478.29 | -0.24 – 0.36 | 0.345 |
| group | 237.21 | 0.13 | -309.95 – 784.37 | -0.18 – 0.44 | 0.393 |
| timepoint [T1] | 81.57 | 0.01 | -312.66 – 475.79 | -0.29 – 0.32 | 0.683 |
| timepoint [T2] | -97.56 | -0.16 | -482.51 – 287.38 | -0.46 – 0.14 | 0.617 |
| health | 67.85 | 0.05 | -152.81 – 288.51 | -0.11 – 0.22 | 0.544 |
| self-efficacy PA | -5.72 | -0.07 | -22.52 – 11.08 | -0.27 – 0.13 | 0.501 |
| M intrinsic PA | 0.18 | 0.00 | -11.13 – 11.50 | -0.20 – 0.20 | 0.975 |
| family health climate PA | 8.19 | 0.07 | -13.76 – 30.13 | -0.12 – 0.27 | 0.462 |
| sex | 338.49 | 0.19 | 98.90 – 578.08 | 0.05 – 0.32 | **0.006** |
| group × timepoint [T1] | -135.30 | -0.08 | -671.33 – 400.74 | -0.38 – 0.23 | 0.618 |
| group × timepoint [T2] | -81.70 | -0.05 | -599.18 – 435.78 | -0.34 – 0.25 | 0.755 |
| **Random Effects** | | | | | |
| σ^2^ | 385291.86 | | | | |
| τ_00_ _family_ | 442431.18 | | | | |
| ICC | 0.53 | | | | |
| N _family_ | 42 | | | | |
| Observations | 140 | | | | |
| Marginal R^2^ / Conditional R^2^ | 0.056 / 0.561 | | | | |

**Table S3**

*Multilevel model analysis for the influence of the three week intervention period on device-based measured physical activity (accelerometry using 10 second epochs) in adults and children. Displayed are the results of the group (control = 0, intervention = 1) x time (dummy coded with T_0_ as reference for T_1_) interaction, and the secondary outcomes self-efficacy, intrinsic motivation, the family health climate (all relating to physical activity), and health status as control variables. Additionally, adult/child (0 = adult, 1 = child), sex (0 = male, 1 = female), and non wear time (nwt) per week are included as a control variables. All results are displayed using the raw estimates (minutes of moderate to vigorous physical activity (MVPA) per week), the standardized Beta (β), 95% confidence intervals (CI), and standardized (std.) 95% CI. Additionally, the within-person variance (σ^2^), the between-person variance (τ_00_* _family_*), the intraclass correlation coefficient (ICC), the number of families (N _family_), the number of observations, and the marginal and conditional R² are displayed.*

|  | **Accelerometry**  **MVPA/week** | | | | |
| --- | --- | --- | --- | --- | --- |
| *Predictors* | *Estimates* | *β* | *CI* | *std. CI* | *p* |
| (Intercept) | 827.63 | 0.07 | 441.72 – 1213.54 | -0.12 – 0.26 | **<0.001** |
| group | 17.20 | 0.03 | -89.70 – 124.10 | -0.16 – 0.22 | 0.751 |
| timepoint [T1] | -54.61 | -0.16 | -150.86 – 41.65 | -0.39 – 0.08 | 0.265 |
| health | 31.43 | 0.08 | -20.31 – 83.16 | -0.05 – 0.22 | 0.232 |
| self-efficacy PA | 3.87 | 0.14 | -0.62 – 8.36 | -0.02 – 0.30 | 0.091 |
| M intrinsic PA | 0.55 | 0.03 | -2.42 – 3.53 | -0.13 – 0.19 | 0.714 |
| family health climate PA | 0.78 | 0.02 | -5.15 – 6.71 | -0.13 – 0.17 | 0.796 |
| adult child | 19.32 | 0.03 | -59.22 – 97.86 | -0.11 – 0.17 | 0.628 |
| sex | -168.57 | -0.30 | -237.30 – -99.84 | -0.42 – -0.18 | **<0.001** |
| nwt | -0.09 | -0.22 | -0.15 – -0.03 | -0.37 – -0.08 | **0.002** |
| group × timepoint [T1] | 20.69 | 0.04 | -112.98 – 154.36 | -0.20 – 0.27 | 0.761 |
| **Random Effects** | | | | | |
| σ^2^ | 57701.05 | | | | |
| τ_00_ _family_ | 10107.31 | | | | |
| ICC | 0.15 | | | | |
| N _family_ | 44 | | | | |
| Observations | 220 | | | | |
| Marginal R^2^ / Conditional R^2^ | 0.184 / 0.306 | | | | |

**Table S4**

*Multilevel model analysis for the influence of the three week intervention period on device-based measured physical activity (accelerometry using 10 second epochs) in adults and children. Displayed are the results of the group (control = 0, intervention = 1) x time (dummy coded with T_0_ as reference for T_1_) interaction, and the secondary outcomes self-efficacy, intrinsic motivation, the family health climate (all relating to physical activity), and health status as control variables. Additionally, adult/child (0 = adult, 1 = child), sex (0 = male, 1 = female), and non wear time (nwt) per week are included as a control variables. All results are displayed using the raw estimates (step count per week), the standardized Beta (β), 95% confidence intervals (CI), and standardized (std.) 95% CI. Additionally, the within-person variance (σ^2^), the between-person variance (τ_00_* _family_*), the intraclass correlation coefficient (ICC), the number of families (N _family_), the number of observations, and the marginal and conditional R² are displayed.*

|  | **Accelerometry**  **step count/week** | | | | |
| --- | --- | --- | --- | --- | --- |
| *Predictors* | *Estimates* | *β* | *CI* | *std. CI* | *p* |
| (Intercept) | 79710.99 | 0.04 | 44900.04 – 114521.94 | -0.18 – 0.26 | **<0.001** |
| group | 7357.35 | 0.15 | -3745.73 – 18460.42 | -0.08 – 0.37 | 0.193 |
| timepoint [T1] | -2947.51 | -0.07 | -11173.24 – 5278.23 | -0.31 – 0.16 | 0.481 |
| health | 2091.32 | 0.06 | -2448.98 – 6631.61 | -0.07 – 0.20 | 0.365 |
| self-efficacy PA | 362.27 | 0.15 | -33.55 – 758.08 | -0.01 – 0.31 | 0.073 |
| M intrinsic PA | 45.91 | 0.03 | -218.17 – 309.99 | -0.13 – 0.19 | 0.732 |
| family health climate PA | -205.46 | -0.06 | -754.72 – 343.80 | -0.22 – 0.10 | 0.462 |
| adult child | -1102.82 | -0.02 | -7866.85 – 5661.20 | -0.16 – 0.11 | 0.748 |
| sex | -2450.69 | -0.05 | -8381.59 – 3480.20 | -0.17 – 0.07 | 0.416 |
| nwt | -9.45 | -0.26 | -14.70 – -4.20 | -0.41 – -0.12 | **<0.001** |
| group × timepoint [T1] | 2066.73 | 0.04 | -9367.88 – 13501.34 | -0.19 – 0.27 | 0.722 |
| **Random Effects** | | | | | |
| σ^2^ | 410702476.77 | | | | |
| τ_00_ _family_ | 184263507.48 | | | | |
| ICC | 0.31 | | | | |
| N _family_ | 44 | | | | |
| Observations | 220 | | | | |
| Marginal R^2^ / Conditional R^2^ | 0.117 / 0.391 | | | | |

**Table S5**

*Multilevel model analysis for the influence of the three week intervention period on self-reported fruit and vegetable intake (single item questionnaire) in adults and children. Displayed are the results of the group (control = 0, intervention = 1) x time (dummy coded with T_0_ as reference for T_1_ and T_2_) interaction, and the secondary outcomes self-efficacy, intrinsic motivation, the family health climate (all relating to healthy eating), and health status as control variables. Additionally, adult/child (0 = adult, 1 = child), and sex (0 = male, 1 = female) are included as a control variables. All results are displayed using the raw estimates (fruit and vegetable portions per week), the standardized Beta (β), 95% confidence intervals (CI), and standardized (std.) 95% CI. Additionally, the within-person variance (σ^2^), the between-person variance (τ_00_* _family_*), the intraclass correlation coefficient (ICC), the number of families (N _family_), the number of observations, and the marginal and conditional R² are displayed.*

|  | **Questionnaire**  **fruit and vegetable intake/week** | | | | | |
| --- | --- | --- | --- | --- | --- | --- |
| *Predictors* | *Estimates* | *β* | *CI* | *std. CI* | *p* | *std. p* |
| (Intercept) | 4.52 | -0.02 | -2.25 – 11.29 | -0.24 – 0.21 | 0.190 | 0.891 |
| group | 5.09 | 0.26 | 0.62 – 9.56 | 0.03 – 0.49 | **0.026** | **0.026** |
| timepoint [T1] | 0.06 | 0.01 | -2.51 – 2.63 | -0.16 – 0.19 | 0.964 | 0.873 |
| timepoint [T2] | 0.95 | 0.03 | -1.63 – 3.53 | -0.14 – 0.21 | 0.471 | 0.697 |
| health | -0.87 | -0.06 | -2.01 – 0.26 | -0.15 – 0.02 | 0.129 | 0.129 |
| self-efficacy NU | 0.09 | 0.10 | -0.00 – 0.18 | -0.00 – 0.20 | 0.061 | 0.061 |
| M intrinsic NU | 0.11 | 0.17 | 0.05 – 0.17 | 0.07 – 0.28 | **0.001** | **0.001** |
| family health climate NU | 0.01 | 0.01 | -0.12 – 0.14 | -0.10 – 0.12 | 0.870 | 0.870 |
| adult child | 1.32 | 0.07 | -0.19 – 2.82 | -0.01 – 0.14 | 0.086 | 0.086 |
| sex | 3.12 | 0.16 | 1.54 – 4.69 | 0.08 – 0.24 | **<0.001** | **<0.001** |
| group × timepoint [T1] | 0.14 | 0.01 | -3.27 – 3.55 | -0.17 – 0.18 | 0.936 | 0.936 |
| group × timepoint [T2] | -1.09 | -0.06 | -4.56 – 2.38 | -0.23 – 0.12 | 0.538 | 0.538 |
| **Random Effects** | | | | | | |
| σ^2^ | 43.00 | | | | | |
| τ_00_ _family_ | 40.80 | | | | | |
| ICC | 0.49 | | | | | |
| N _family_ | 44 | | | | | |
| Observations | 356 | | | | | |
| Marginal R^2^ / Conditional R^2^ | 0.168 / 0.573 | | | | | |

**Table S6**

*Multilevel model analysis for the influence of the three week intervention period on self-reported fruit and vegetable intake (diary) in adults and children. Displayed are the results of the group (control = 0, intervention = 1) x time (dummy coded with T_0_ as reference for T_1_) interaction, and the secondary outcomes self-efficacy, intrinsic motivation, the family health climate (all relating to healthy eating), and health status as control variables. Additionally, adult/child (0 = adult, 1 = child), and sex (0 = male, 1 = female) are included as control variables. All results are displayed using the raw estimates (fruit and vegetable portions per week), the standardized Beta (β), 95% confidence intervals (CI), and standardized (std.) 95% CI. Additionally, the within-person variance (σ^2^), the between-person variance (τ_00_* _family_*), the intraclass correlation coefficient (ICC), the number of families (N _family_), the number of observations, and the marginal and conditional R² are displayed.*

|  | **Diary**  **fruit and vegetable intake/week** | | | | |
| --- | --- | --- | --- | --- | --- |
| *Predictors* | *Estimates* | *β* | *CI* | *std. CI* | *p* |
| (Intercept) | 2.61 | -0.01 | -5.42 – 10.64 | -0.24 – 0.23 | 0.523 |
| group | 3.27 | 0.16 | -1.45 – 7.99 | -0.07 – 0.40 | 0.174 |
| timepoint [T1] | -0.60 | 0.01 | -3.13 – 1.94 | -0.16 – 0.18 | 0.644 |
| health | 0.67 | 0.05 | -0.73 – 2.07 | -0.05 – 0.15 | 0.345 |
| self-efficacy NU | 0.06 | 0.06 | -0.06 – 0.18 | -0.07 – 0.19 | 0.348 |
| M intrinsic | 0.16 | 0.25 | 0.07 – 0.24 | 0.11 – 0.38 | **<0.001** |
| family health climate NU | -0.06 | -0.05 | -0.23 – 0.10 | -0.18 – 0.08 | 0.466 |
| adult child NU | 1.01 | 0.05 | -0.78 – 2.80 | -0.04 – 0.14 | 0.269 |
| sex | 4.90 | 0.24 | 3.00 – 6.80 | 0.15 – 0.34 | **<0.001** |
| group × timepoint [T1] | 1.25 | 0.06 | -2.23 – 4.72 | -0.11 – 0.24 | 0.481 |
| **Random Effects** | | | | | |
| σ^2^ | 42.16 | | | | |
| τ_00_ _family_ | 44.76 | | | | |
| ICC | 0.51 | | | | |
| N _family_ | 42 | | | | |
| Observations | 238 | | | | |
| Marginal R^2^ / Conditional R^2^ | 0.205 / 0.614 | | | | |
